# Regulation of lung progenitor plasticity and repair by fatty acid oxidation

**DOI:** 10.1172/jci.insight.165837

**Published:** 2025-02-10

**Authors:** Quetzalli D. Angeles-Lopez, Jhonny Rodriguez-Lopez, Paula Agudelo Garcia, Jazmin Calyeca, Diana Álvarez, Marta Bueno, Lan N. Tu, Myriam Salazar-Terreros, Natalia Vanegas-Avendaño, Jordan E. Krull, Aigul Moldobaeva, Srimathi Bogamuwa, Stephanie S. Scott, Victor Peters, Brenda F. Reader, Sruti Shiva, Michael Jurczak, Mahboobe Ghaedi, Qin Ma, Toren Finkel, Mauricio Rojas, Ana L. Mora

**Affiliations:** 1 Department of Internal Medicine, Division of Pulmonary Critical Care and Sleep Medicine, The Ohio State University, Columbus, Ohio, USA.; 2 Aging Institute, Department of Medicine, and; 3 Department of Medicine, Division of Pulmonary, Allergy, Critical Care and Sleep Medicine, University of Pittsburgh, Pittsburgh, Pennsylvania, USA.; 4 Department of Biomedical Informatics, College of Medicine, and; 5 Pelotonia Institute for Immuno-Oncology, James Comprehensive Cancer Center, The Ohio State University, Columbus, Ohio, USA.; 6 Bioscience COPD/IPF, Research and Early Development, Respiratory & Immunology, BioPharmaceuticals R&D, AstraZeneca, Gaithersburg, Maryland, USA.; 7 Department of Surgery, Division of Transplantation Surgery, The Ohio State University, Columbus, Ohio, USA.; 8 Department of Medicine, Center for Metabolism and Mitochondrial Medicine, and; 9 Department of Pharmacology and Chemical Biology, Vascular Medicine Institute, University of Pittsburgh, Pittsburgh, Pennsylvania, USA.

**Keywords:** Metabolism, Pulmonology, Fatty acid oxidation, Fibrosis, Mitochondria

## Abstract

Idiopathic pulmonary fibrosis (IPF) is an age-related interstitial lung disease, characterized by inadequate alveolar regeneration and ectopic bronchiolization. While some molecular pathways regulating lung progenitor cells have been described, the role of metabolic pathways in alveolar regeneration is poorly understood. We report that expression of fatty acid oxidation (FAO) genes is significantly diminished in alveolar epithelial cells of IPF lungs by single-cell RNA sequencing and tissue staining. Genetic and pharmacological inhibition in AT2 cells of carnitine palmitoyltransferase 1a (CPT1a), the rate-limiting enzyme of FAO, promoted mitochondrial dysfunction and acquisition of aberrant intermediate states expressing basaloid, and airway secretory cell markers SCGB1A1 and SCGB3A2. Furthermore, mice with deficiency of CPT1a in AT2 cells show enhanced susceptibility to developing lung fibrosis with an accumulation of epithelial cells expressing markers of intermediate cells, airway secretory cells, and senescence. We found that deficiency of CPT1a causes a decrease in SMAD7 protein levels and TGF-β signaling pathway activation. These findings suggest that the mitochondrial FAO metabolic pathway contributes to the regulation of lung progenitor cell repair responses and deficiency of FAO contributes to aberrant lung repair and the development of lung fibrosis.

## Abstract 2

Fatty acid oxidation is required for succesful alveolar progenitor differentiation, loss of FAO leads to the accumulation of intermediate cell states and failure to repair the alveoli.

## Graphical abstract

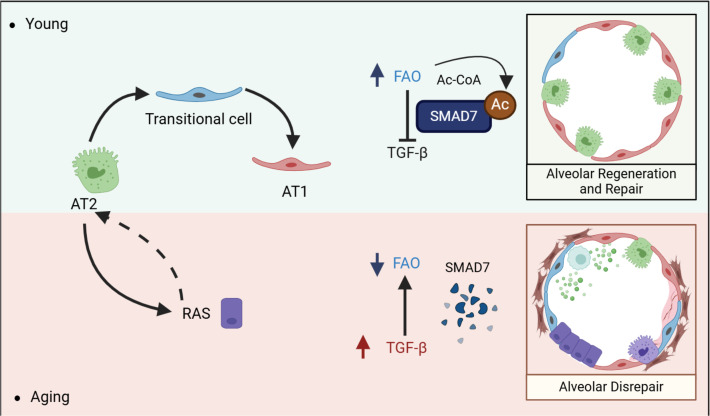

## Introduction

Maintenance of tissue fidelity and function after injury depends on the ability of progenitor cells to engage in the proper differentiation program. While turnover and proliferation are not highly active in the mature lung, the lung has a remarkable capacity to repair after injury. A subset of Axin2^+^ alveolar type 2 (AT2) cells act as progenitors of both AT2 and alveolar type 1 (AT1) cell populations. In lungs with idiopathic pulmonary fibrosis (IPF), decreased AT2 stemness is associated with diminution of AT1 and AT2-AT1 transitional state cells and the appearance of an aberrant epithelial cell population characterized by the expression of airway epithelial marker keratin 17 (KRT17), but negative for the basal cell marker KRT5. KRT17^+^KRT5^–^ cells express extracellular matrix (ECM) components, cell adhesion, motility program, TGF-β1 target genes, and senescence markers. Additionally, new populations of progenitor cells, called respiratory alveolar secretory (RAS) cells, have been reported in human bronchioles. These cells express airway secretory (SCGB3A1 and SCGB1A1) and AT2 (surfactant protein C [SP-C], SP-B) cell markers, and can generate both airway and alveolar epithelial lineages (1). Additionally, studies using a nonhuman primate in vivo model of lung injury and human organoids showed that AT2 cells can transiently acquire a bipotent progenitor state termed AT0 with the capacity to differentiate into AT1 or RAS cells, also denominated terminal and bronchiolar secretory cells (2). RAS cells have been reported in IPF lungs, other fibrotic interstitial lung diseases, and severe lung injuries (2, 3). Importantly, the accumulation of KRT17^+^ basaloid and RAS cells is associated with aberrant activity of pathways known to regulate AT2 cell differentiation and exacerbate profibrotic responses and lung remodeling.

In mouse models of fibrosis, single-cell RNA-sequencing (scRNA-seq) studies have identified AT2 transitional cells, called alveolar differentiation intermediate (ADI) cells, which strongly resemble the gene expression signature of *Krt17^+^* *Krt5^–^* cells with high *Krt8* expression (4–6). ADIs correspond to human KRT17^+^KRT5^–^ cells, a unique transitional cell population with enrichment in TGF-β, TP53, YAP, and Wnt pathways and increased expression of transcripts associated with cell cycle arrest and senescence. Although bronchiolar structures are absent in mice, an airway distal stem cell population called bronchioalveolar stem cells (BASCs) has been identified. These cells express Scgb1a1 and Sftpc and function as progenitor cells of both airway and alveolar epithelial lineages after severe lung injury (7–9).

**Figure 1:**
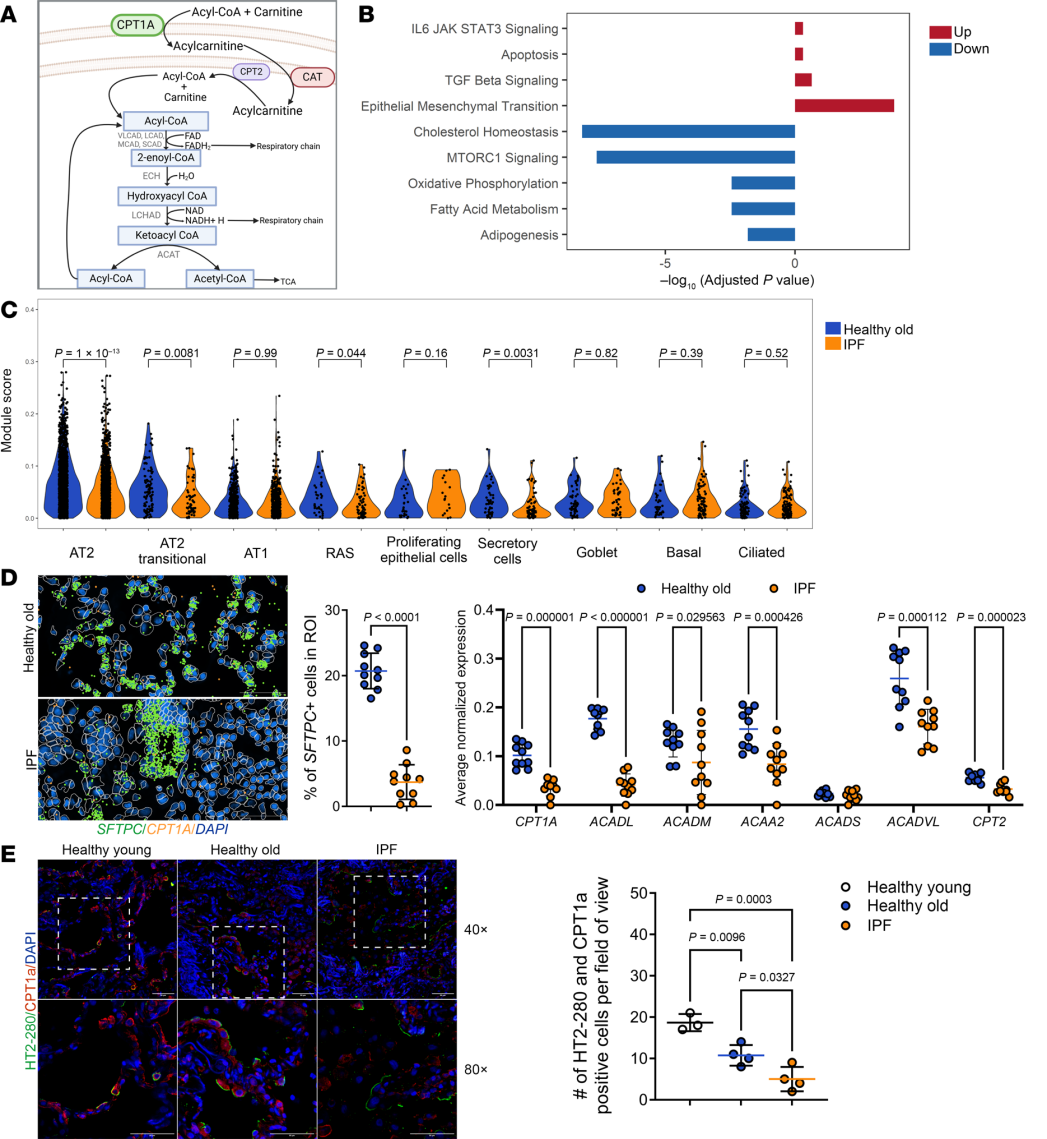
FAO gene expression decreases with age and IPF disease in lung epithelial cells. (**A**) Fatty acid oxidation (FAO) pathway. (**B**) Up- (red) and downregulated (blue) pathways between healthy donor (*n* = 9) and IPF (*n* = 6) AT2 cells (adjusted *P* < 0.05). (**C**) FAO score of all the epithelial and airway subtypes from healthy old deceased donor (*n* = 9) and IPF lungs (*n* = 6). Violin plots display the distribution of data, and statistical significance was determined by Wilcoxon’s test. (**D**) Visualization and quantification of lung transcriptomic information obtained through Xenium-based spatial transcriptomics analysis. In the image, colors correspond to the legend. Each dot represents a sequencing spot. Scale bar: 50 μm. Left graph shows the decrease in SFTPC^+^ cells in IPF lung tissue and right graph shows the decrease in transcripts related to the FAO pathway in SFTPC^+^ cells in healthy old donors (*n* = 2) and IPF (*n* = 2). Data represent mean ± SD; each dot represents a field of view (FOV) from 2 tissues per condition. Statistical significance was determined by 2-tailed Student’s *t* test (left graph) and individual unpaired *t* test (right graph). (**E**) Immunofluorescent staining showing CPT1a expression (red) in AT2 cells (green) in human lungs from young and old deceased donor or IPF lungs (*n* = 3, per group) and quantification of number of HT2-280^+^ and CPT1a^+^ cells. Scale bars: 50 μm. Data represent mean ± SD; each dot represents a FOV. Statistical significance was determined by 1-way ANOVA followed by Tukey’s multiple-comparison test.

Metabolic changes are currently recognized as one of the hallmarks of aging and fibrosis across many organs (10). Our group has contributed to the characterization of mitochondrial dysfunction as a critical factor in the increased age-related susceptibility to lung fibrosis. An important mitochondrial metabolic pathway is fatty acid oxidation (FAO), a multistep process of breaking down fatty acids (FAs) into acetyl-coenzyme A (acetyl-CoA) units. The first step is the import of fatty acyl-CoAs into mitochondria by the carnitine palmitoyltransferase I (CPT1)-CPT2 system, which is the rate-limiting factor of the FAO metabolic pathway. Acyl-CoAs are then metabolized by membrane-bound enzymes, including very long-, long-, medium-, and short-chain acyl-CoA dehydrogenases. FAO-generated acetyl-CoA is one of the main substrates for protein acetylation and serves as a critical metabolite in the adaptation to cell stress responses (11). In addition, FAO is interrelated with the electron transport chain and the tricarboxylic acid cycle, both of which contribute to mitochondrial homeostasis. (Figure 1A). With increasing age, a significant decline in FAO has been documented in various human tissues (12–14). Furthermore, a metabolic shift involving FAO has been found to be critical in defining cell fate in several physiological and pathological conditions, including endothelial-mesenchymal transition, immune cell activation, and airway epithelial cell differentiation (15–19).

TGF-β signaling plays a critical role during regeneration of damaged alveolar epithelial cells after injury, and is highly activated in AT2 cells during early states of differentiation. However, at later differentiation states, TGF-β is deactivated followed by the full activation of the AT1 program (20). TGF-β has also been shown to promote transdifferentiation of human AT2 cells into basal cells (21). Here, we have found a role for FAO in regulating alveolar lung repair. We show that FAO inhibits TGF-β signaling through the modulation of acetyl-CoA levels and the total protein levels of the TGF-β negative regulator SMAD7. Genetic and pharmacological disruption of carnitine palmitoyltransferase 1a (CPT1a) modulates in vitro and in vivo AT2 differentiation by promoting accumulation of aberrant basaloid and airway secretory intermediate cells and reducing the resistance to developing lung fibrosis after injury. Altogether, our study establishes that FAO in AT2 cells is an important regulator of differentiation programs and alveolar regeneration.

## Results

### Progenitor AT2 cells from fibrotic lungs have defective FAO gene expression.

We performed scRNA-seq analysis in human lung samples from healthy old donors (>58 years old; *n* = 9) and IPF explants (55–71 years old; *n* = 6) (Supplemental Tables 1 and 2). IPF samples were collected from the lower lobe of the lung to capture changes associated with severe disease (Supplemental Figure 1A).

Our previous work shows that dysfunctional mitochondria significantly accumulate in AT2 cells from old and IPF lungs (22, 23). Comparison of scRNA-seq data between age-matched healthy and IPF AT2 epithelial cells showed that top enriched pathways among differentially expressed genes were related to lipid and mitochondrial metabolism (Figure 1B). Studies using BODIPY staining revealed increased lipid inclusions in AT2 cells of IPF lungs (Supplemental Figure 1B). Lipid droplets are the primary source of FAs used for the catabolic process of β-oxidation that produces acetyl-CoA, and low degradation or accumulation of these lipid inclusions is associated with deficient FAO. Notably, impaired FAO is recognized to play a critical role in epithelial differentiation and repair (19, 24). To determine whether changes in the expression of FAO enzymes occur in lung epithelial cells during aging and IPF, we analyzed the expression of FAO genes. We found that AT2 cells from IPF lungs have lower expression of several FAO genes in comparison with cells from healthy old lungs, including *CPT1a*, *ACADL*, and *ACAT1* (Supplemental Figure 1, C and D). Calculation of a high composite FAO gene score revealed that the expression of FAO genes decreased from healthy old to IPF lungs in AT2, AT2 transitional, RAS (*SCGB1A1*^+^ *SCGB3A2*^+^ *SFTPB*^+^, low *SPC*), and secretory (*SCGB1A1*^+^ *SCGB3A2*^+^ *SFTPC*^–^) cell populations in IPF lungs in comparison with healthy old lungs (Figure 1C and Supplemental Table 3).

In addition, spatial transcriptomic studies were performed in healthy and IPF lungs to analyze in situ expression of FAO genes at the single-cell level using the Xenium 10X platform. These studies confirmed a reduced percentage of SFTPC^+^ cells in IPF lungs (Figure 1D). Furthermore, in situ expression of FAO genes, including *CPT1A*, *ACADL*, *ACADM*, *ACAA2*, *ACADVL*, and *CPT2*, was significantly diminished in SFTPC^+^ cells from IPF lungs (Figure 1D). To validate our findings, we performed immunofluorescence on young, old, and IPF lungs using antibodies against the mitochondrial protein CPT1a, the rate-limiting enzyme of FAO, and HT2-280 as a marker of AT2 cells. Interestingly, we found that the expression of CPT1a at the protein level was lower in old compared with young donors and was further diminished in HT2-280^+^ cells from IPF lungs (Figure 1E). To further corroborate that FAO decreases with aging in lung epithelial cells, we assessed FAO by measuring the ability to oxidize ^14^C-palmitate (a 16-carbon long-chain FA), measuring the radioactivity incorporated into acid-soluble metabolites. Our data show that aging lung epithelial cells (EpCAM^+^) from mice have a significantly decreased ability to oxidize FAs (Supplemental Figure 1E).

Altogether, our data show that expression of genes associated with FAO metabolic pathway and FAO activity is reduced with age in lung epithelial cells. In addition, reduced expression of FAO genes was found in intermediate-state epithelial lung cell populations that coexpress markers of AT2 and airway cells and accumulate in IPF lungs.

### Inhibition of FAO in AT2 cells induces the emergence of basaloid and secretory cells.

**Figure 2:**
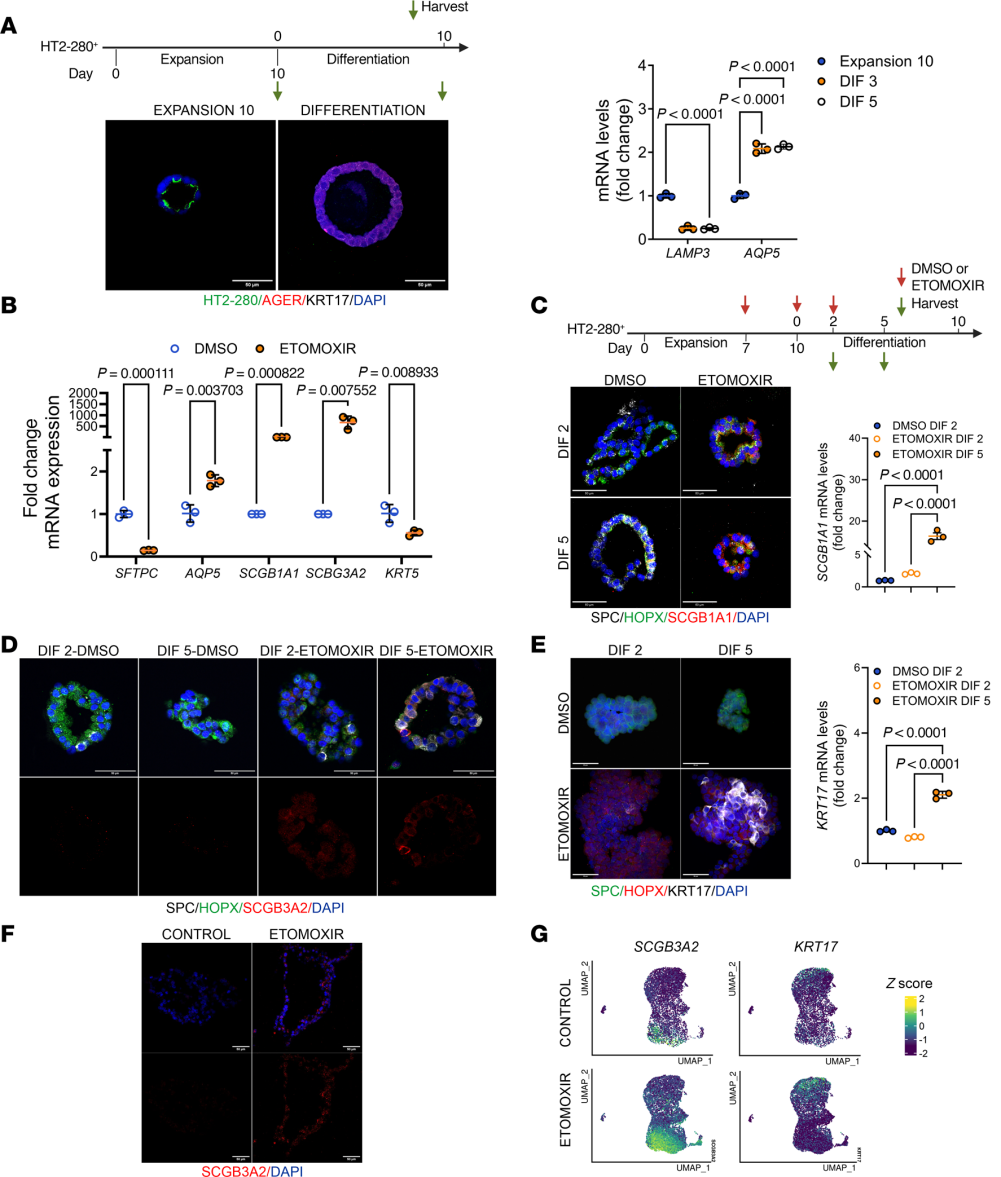
Inhibition of CPT1a in AT2 cells induces the emergence of RAS and basaloid cell phenotypes in human organoids. (**A**) Top: Timeline of the experiment. Bottom: Immunofluorescence depicting expression of AGER and KRT17 in human HT2-derived organoids during the expansion and differentiation phases and mRNA levels of LAMP3 and AQP5 (*n* = 3). Data represent mean ± SD; each dot represents a technical replicate. Statistical significance was determined by 2-way ANOVA followed by Šidák’s multiple-comparison test. (**B**) mRNA levels of epithelial and secretory markers in organoids on day 10 of expansion treated with DMSO or etomoxir. Data represent mean ± SD; each dot represents a technical replicate. Statistical significance was determined by multiple unpaired *t* test. (**C**) Top: Scheme of the human alveolar organoid experimental design in the presence of CPT1a inhibitor etomoxir. Bottom: Representative immunofluorescence images (left) and mRNA levels (right) showing that SCGB1A1 expression increases when CPT1a is pharmacologically inhibited with etomoxir (*n* = 3, each group). Data represent mean ± SD; each dot represent a technical replicate. Statistical significance was determined by 1-way ANOVA followed by Tukey’s multiple-comparison test. (**D**) Expression of SCGB3A2 in organoids treated with etomoxir. SP-C (white) was used as an AT2 marker, SCGB3A2 (red) was used as a secretory cell marker, and HOPX (green) was used as an AT1 marker. (**E**) Scheme of the human alveolar organoid experimental setup with CPT1a inhibitor treatment. Representative immunofluorescence images and mRNA levels showing increased KRT17 expression upon CPT1a pharmacological inhibition with etomoxir (*n* = 3, per condition). SP-C (green) was used as an AT2 marker, HOPX (red) was used as an AT1 marker, and KRT17 (white) was used as a transitional cell marker. Data represent mean ± SD: each dot represents a technical replicate. Statistical significance was determined by 1-way ANOVA followed by Tukey’s multiple-comparison test. (**F**) Expression of SCGB3A2 in organoids from iAT2 cells treated with etomoxir. (**G**) UMAP showing the expression of *SCGB3A2* and *KRT17* in organoids from iAT2 cells culture in CK-DCI media. Color scale denotes the normalized expression. All scale bars: 50 μm.

To better understand the connection between AT2 differentiation and the FAO metabolic pathway, we analyzed 3D organoids derived from isolated primary HT2-280^+^ AT2 cells from healthy adult donor human lungs. These organoids were cultured under chemically defined conditions to support the expansion and differentiation of human AT2 cells (25). Using a protocol of 10 days of expansion followed by 10 days of differentiation, we observed changes in the expression of AT2 and AT1 markers (Figure 2A). During the expansion phase, treatment with the CPT1a inhibitor etomoxir, compared with the vehicle, reduced transcript levels of *SFTPC* (Figure 2B). We observed elevated expression of AT1 marker *AQP5* and a dramatic increase in secretory marker *SCGB1A1* and *SCGB3A2* (Figure 2B). Then, we analyzed the effect of etomoxir-mediated FAO inhibition between day 7 of expansion and day 2 and 5 of differentiation. We found that etomoxir treatment induced the expression of both SCGB1A1 and SCGB3A on day 2 and 5 of differentiation (Figure 2, C and D). Increased expression of *SCGB1A1* was confirmed by qPCR on day 5 after differentiation in etomoxir-treated samples (Figure 2C). Next, we evaluated whether etomoxir treatment induced KRT17, a marker for alveolar intermediate basaloid cells. Expression of KRT17 was observed only in etomoxir-treated organoids on day 5 after differentiation (Figure 2E). *KRT17* transcript levels were confirmed by qRT-PCR (Figure 2E).

To further verify the impact of inhibition of FAO in AT2 cells, we analyzed the induced pluripotent stem cell–derived AT2 (iAT2) model system (Supplemental Figure 2A) (26). Organoids derived from iAT2 cells cultured in CK-DCI media (composed of CHIR99021, keratinocyte growth factor, dexamethasone, cAMP, and 3-isobutyl-1-methylxanthine) (27) and in the presence of the CPT1a inhibitor etomoxir expressed SCGB3A2 and KRT17 by immunofluorescent staining (Figure 2F and Supplemental Figure 2B). We further performed scRNA-seq analysis in iAT2 organoids (Supplemental Table 4). Using a uniform manifold approximation and projection (UMAP) representation, we identified iAT2 cells expressing higher levels of *SCGB3A2* and *KRT17* in the presence of etomoxir (Figure 2G and Supplemental Figure 2C). Together, these results suggest that inhibition of FAO promotes the expression of markers of basaloid and RAS cells.

### In vivo Cpt1a loss in AT2 cells increases susceptibility to lung fibrosis after injury.

**Figure 3:**
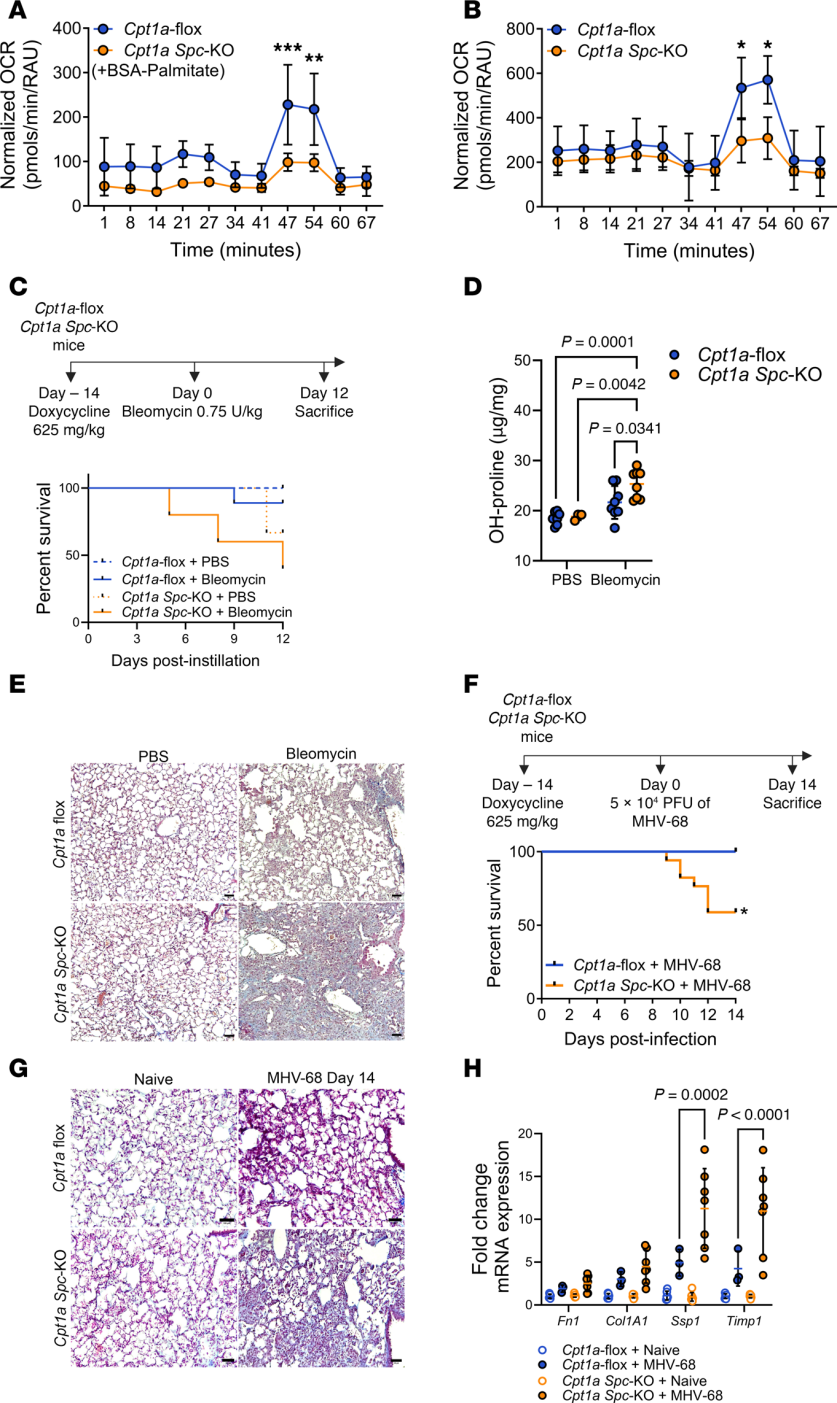
*Cpt1a* deletion in AT2 cells reduces resistance to lung fibrosis. (**A** and **B**) Oxygen consumption rate (OCR) in EpCAM^+^ cells from floxed and *Cpt1a Spc*-KO mice (*n* = 3, per genotype) showing BSA-palmitate–challenged (**A**) and baseline cells (**B**). RAU, relative absorbance units. Data represent mean ± SD; statistical significance was determined by 2-way repeated-measures ANOVA. *P < 0.05, **P < 0.01, ***P < 0.001. (**C**) Scheme of the experiment and Kaplan-Meier curve of *Cpt1a*-floxed and *Cpt1a Spc*-KO mice upon bleomycin injury (*n* = 6–10, starting mice). (**D**) Hydroxyproline (OH-proline) lung content was determined in *Cpt1a*-floxed and *Cpt1a Spc*-KO mice, showing an increase in collagen deposition in *Cpt1a Spc*-KO lungs after bleomycin injury (*Cpt1a*-floxed-PBS, *n* = 8; *Cpt1a Spc*-KO-PBS, *n* = 3; *Cpt1a*-floxed-Bleomycin, *n* = 8; *Cpt1a Spc*-KO-Bleomycin, *n* = 8). Data represent mean ± SD: statistical significance was determined by 2-way ANOVA followed by Tukey’s multiple-comparison test. (**E**) Representative Masson’s trichrome micrographs 12 days after bleomycin injury showing collagen deposition (blue) in lungs from *Cpt1a Spc*-KO and floxed mice. Scale bars: 50 μm. (**F**) Scheme of the experiment and Kaplan-Meier curve of MHV-68–infected floxed and *Cpt1a Spc*-KO mice (*n* = 3–6, starting mice). (**G**) Representative Masson’s trichrome micrographs of lungs from *Cpt1a Spc*-KO and floxed mice 14 days after MHV-68 infection showing collagen deposition (blue). Scale bars: 50 μm. (**H**) mRNA levels of senescence and fibrosis markers in EpCAM^+^ epithelial cells from floxed (*n* = 6) and *Cpt1a Spc*-KO (*n* = 7) naive mice and floxed (*n* = 3) and *Cpt1a Spc*-KO (*n* = 7) infected mice. Data represent mean ± SD. Statistical significance was determined by 2-way ANOVA followed by Tukey’s multiple-comparison test.

To further understand the physiological consequences of FAO deficiency in AT2 cells, we developed an inducible conditional AT2 cell *Cpt1a*-knockout (*Cpt1a*-KO) mouse under the control of the SP-C promoter (*Cpt1a Spc*-KO). Efficient KO of *Cpt1a* was assessed by qPCR analysis in EpCAM^+^ cells isolated from lungs (Supplemental Figure 3A). Impaired FAO in *Cpt1a Spc*-KO mice was validated using a palmitate oxidation stress test in freshly isolated primary AT2 cells. BSA-palmitate–challenged lung epithelial cells from *Cpt1a Spc*-KO mice exhibited defective FAO, as assessed by their lower oxygen consumption rate (OCR) compared with *Cpt1a*-floxed controls (Figure 3A). We further assessed mitochondrial function in primary AT2 *Cpt1a*-KO cells and found that, overall, KO cells exhibited lower mitochondrial respiration (Figure 3B).

Next, focusing on the potential role of FAO in enhancing the resistance to lung injury and promoting repair responses, we challenged *Cpt1a*-floxed and *Cpt1a Spc*-KO mice with bleomycin by oropharyngeal administration (Figure 3C). We found that KO mice exhibited increased clinical severity, with more than 50% mortality (Figure 3C) and greater weight loss (Supplemental Figure 3B). Lungs of *Cpt1a* *Spc*-KO mice showed a remarkable increase in collagen deposition and fibrosis, as measured by hydroxyproline levels and trichrome staining of lung sections (Figure 3, D and E). To validate our observations, we used a second model of fibrosis independent of a genotoxic agent. We challenged *Cpt1a*-floxed and *Cpt1a Spc*-KO mice intranasally with the murine gamma herpesvirus-68 (MHV-68) (28) (Figure 3F). Infected KO mice exhibited a significantly greater mortality and weight loss when compared with infected *Cpt1a*-floxed mice (Figure 3F and Supplemental Figure 3C). CPT1a status did not alter viral load, as observed by qPCR copy number quantification of the viral replication and transcriptional activator ORF50 (Supplemental Figure 3D). As expected, MHV-68–infected *Cpt1a Spc*-KO mice showed augmented signs of fibrosis as evidenced by increased collagen deposition (Figure 3G), a higher fibrosis score (Supplemental Figure 3E), and higher expression of fibrotic markers (Figure 3H). Collectively, our data demonstrate that in vivo deficiency of *Cpt1a* in AT2 cells leads to a higher susceptibility to developing lung fibrosis after injury.

### Loss of Cpt1a promotes the emergence of lung epithelial cells with an ADI phenotype in vivo.

**Figure 4:**
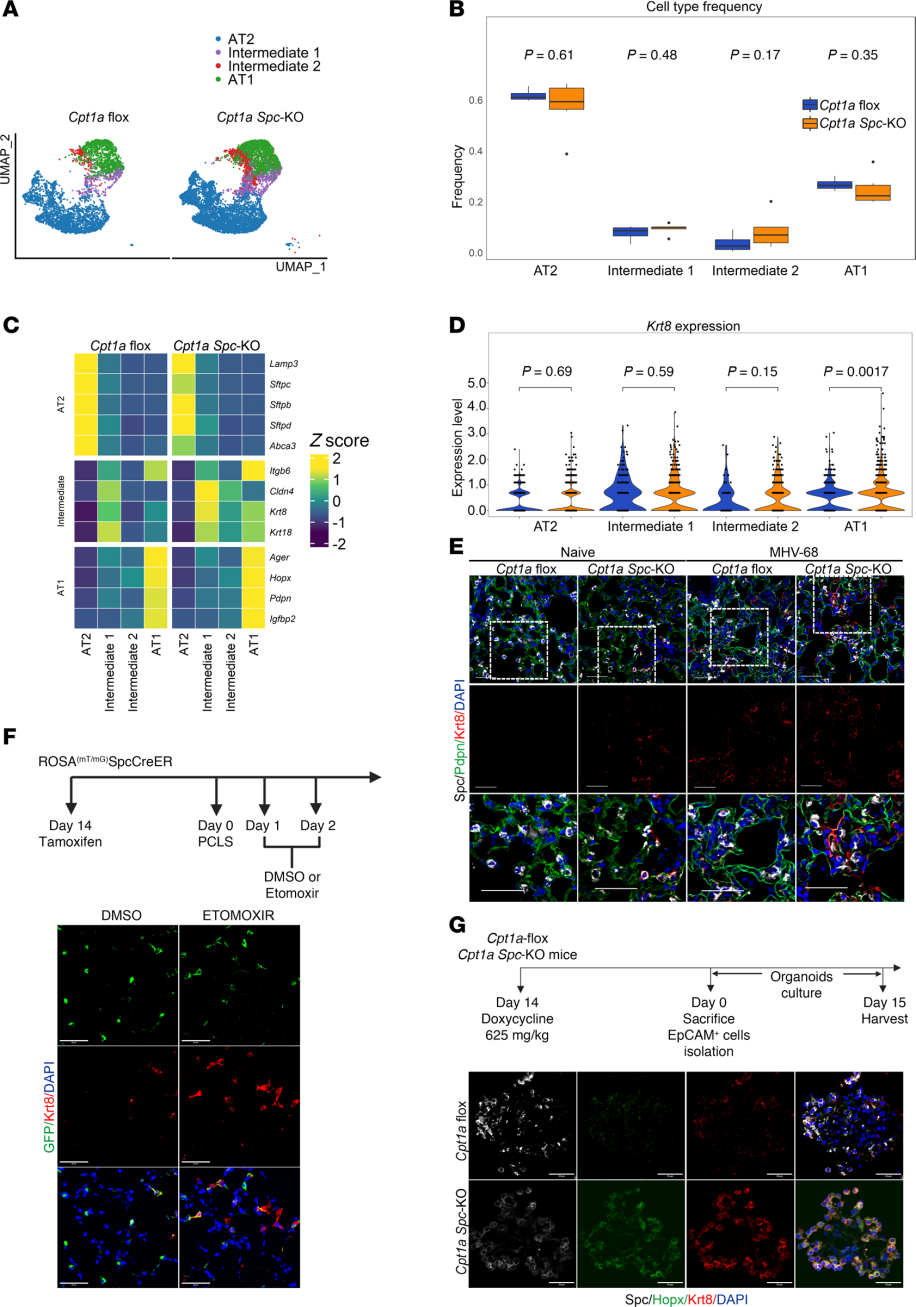
*Cpt1a* deficiency in AT2 cells induces the emergence of cells in an ADI phenotype in vivo and in vitro. (**A**) UMAP shows the distribution of all cell types from MHV-68–infected *Cpt1a Spc*-KO (*n* = 6) and *Cpt1a*-floxed control (*n* = 4) mice. (**B**) Box-and-whisker plots showing cell proportions of subject-specific epithelial cell types in MHV-68–infected *Cpt1a Spc*-KO (*n* = 6) and *Cpt1a*-floxed (*n* = 4) mice. Statistical significance was determined by Wilcoxon’s test. Each box represents the interquartile range (IQR), with the line inside indicating the median frequency. Whiskers extend to the minimum and maximum values within 1.5 times the IQR. Outliers are not explicitly visualized in the plot. (**C**) Heatmap showing *z* values of the mean expression for canonical epithelial markers and differentially expressed genes in each epithelial cell population from MHV-68–infected *Cpt1a*-floxed (*n* = 4) and *Cpt1a Spc*-KO (*n* = 6) mice. (**D**) Violin plots depicting the expression of *Krt8* in the different epithelial cell types in MHV-68–infected *Cpt1a Spc*-KO (*n* = 6) and *Cpt1a*-floxed (*n* = 4) mice. Statistical significance was determined by Wilcoxon’s test. (**E**) Representative immunofluorescence images of KRT8^+^ cells in naive or MHV-68–infected *Cpt1a*-floxed and *Cpt1a Spc*-KO mice (*n* = 4, each group). SP-C (white) was used as an AT2 marker, PDPN (green) was used as an AT2 marker, and KRT8 (red) was used as a transitional cell marker. (**F**) Top: Scheme of the experiment. Bottom: Representative immunofluorescence images of precision-cut lung slices (PCLSs) from ROSA^mT/mG^ SPC-Cre-ER mice treated with vehicle or CPT1a inhibitor (*n* = 5, per condition), showing an increase in KRT8 (red) in GFP^+^ cells after etomoxir treatment. (**G**) Top: Scheme of the mouse organoid culture experimental setup. Bottom: Representative immunofluorescence images of organoids from *Cpt1a*-floxed and *Cpt1a Spc*-KO mice, showing an increase in KRT8 in *Cpt1a Spc*-KO mouse organoids (*n* = 3, each group). SP-C (white) was used as an AT2 marker, HOPX (green) was used as an AT1 marker, and KRT8 (red) was used as a transitional cell marker. All scale bars: 50 μm.

Given the potential role of FAO in AT2 cell–mediated alveolar repair, we performed scRNA-seq in lungs isolated from both *Cpt1a Spc*-KO and *Cpt1a*-floxed mice exposed to MHV-68, and we confirmed that only the cells from *Cpt1a*-floxed mice expressed *Cpt1a* (Supplemental Figure 4A). Using a UMAP representation, we identified epithelial, endothelial, mesenchymal, and immune cell types in the whole lung (Supplemental Figure 4B). A deeper analysis of the alveolar epithelial cell cluster showed 2 intermediate populations (Figure 4, A and B, and Supplemental Table 5). The Intermediate 1 subpopulation has several transcriptional features of ADI cells (*Krt8*, *Krt18*, *Itgb6*, and *Cldn4*) (Figure 4C). These features have been described in diverse conditions, including mouse models of lung injury (29–31). *Krt8* expression was significantly increased in all alveolar cell clusters in the injured *Cpt1a Spc-*KO mice, including AT1 cells (Figure 4D). However, AT1 cells in *Cpt1a* *Spc*-KO mice expressed higher levels of *Igfbp2*, a marker of terminally differentiated AT1 cells (Supplemental Figure 4B) (32). To confirm the preferential emergence of ADI cells in *Cpt1a Spc*-KO mouse lungs, we performed immunofluorescence assays. Strikingly, we found that KO mice spontaneously exhibited intermediate Krt8^+^ cells in the interstitium of uninjured lungs, with a higher accumulation of these cells upon virus-induced injury (Figure 4E and Supplemental Figure 4D). To validate the finding that inhibition of FAO promotes the emergence of a Krt8^+^ ADI state, we used precision-cut lung slices (PCLSs) from ROSA^mT/mG^ SPC-Cre-ER mice that allow for the lineage tracing of AT2 cells (33, 34). PCLSs obtained after in vivo tamoxifen-induced recombination were treated with the CPT1a inhibitor etomoxir on day 1, and harvested on day 2. Compared with DMSO-treated PCLSs, we observed high expression of Krt8 in GFP^+^ AT2 cells after etomoxir treatment (Figure 4F and Supplemental Figure 4E). To determine whether the impaired AT2 differentiation observed in *Cpt1a Spc*-KO mice and PCLSs was due to autocrine signaling, we performed studies in 3D organoid cultures of AT2 cells from *Cpt1a*-floxed control and *Cpt1a Spc*-KO mice, cocultured with lung stromal cells after in vivo doxycycline-induced recombination. CPT1a expression was observed only in 3D organoids derived from epithelial cells from *Cpt1a*-floxed mice, but not in organoids from *Cpt1a Spc*-KO mice (Supplemental Figure 4F). Compared with AT2 organoids derived from *Cpt1a-*floxed control mice, organoids from *Cpt1a Spc*-KO mice showed spontaneous coexpression of markers of AT2 (SP-C), AT1 (HOPX), and ADI cells (Krt8) (Figure 4G and Supplemental Figure 4G). These results indicate that deficiency of CPT1a alters AT2 differentiation and induces the accumulation of an ADI-state cell population.

### Deficiency of CPT1a in AT2 cells promotes a RAS phenotype in alveolar cells.

**Figure 5:**
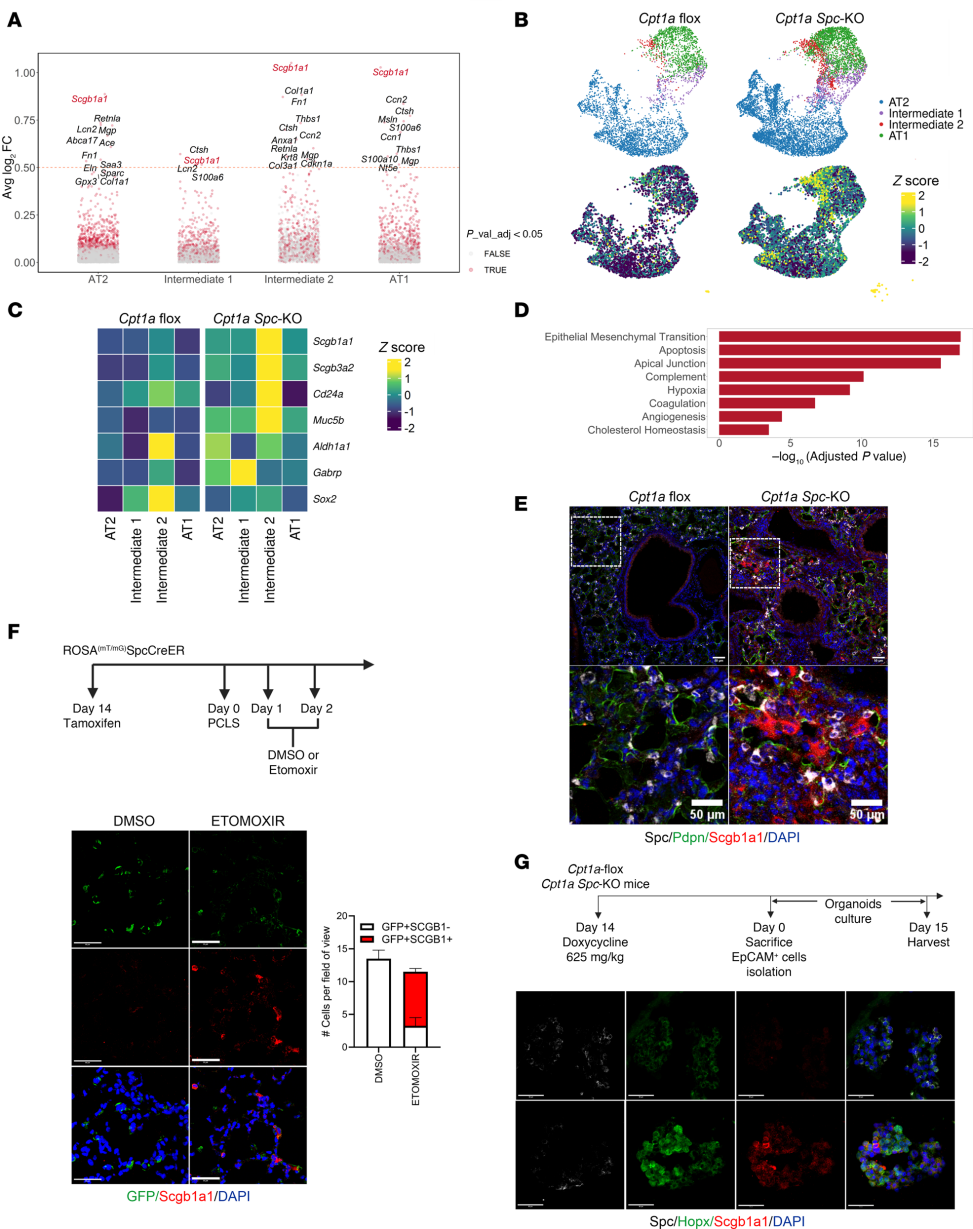
*Cpt1a* deficiency promotes a RAS intermediate phenotype. (**A**) Upregulated genes in *Cpt1a Spc*-KO vs. *Cpt1a*-floxed mice by epithelial cell type. Red color denotes significantly upregulated genes. (**B**) UMAP shows the distribution of all cell types and expression of *Scgb1a1* in lung epithelial cells from MHV-68–infected *Cpt1a Spc*-KO (*n* = 6) and *Cpt1a*-floxed (*n* = 4) mice. (**C**) Heatmap showing *z* values of the mean expression for canonical epithelial markers and differentially expressed genes in AT1, Intermediate 1, Intermediate 2, and AT1 cell types, alongside basal cell markers. (**D**) Enrichment pathway analysis of Intermediate 2 cell population in *Cpt1a Spc*-KO mice. (**E**) Representative immunofluorescence images of the lungs of MHV-68–infected *Cpt1a*-floxed (*n* = 4) and *Cpt1a Spc*-KO (*n* = 6) mice. SP-C (white) was used as an AT2 marker, SCGB1 (red) was used as a secretory cell marker, and PDPN (green) was used as an AT1 marker. (**F**) Top: Scheme of the experiment. Bottom: Immunofluorescent staining and quantification of PCLSs from lineage tracing ROSA^mT/mG^ SPC-Cre-ER mice treated with PBS or etomoxir, depicting higher SCGB1A1 intensity under etomoxir treatment. Data represent mean ± SD, *n* = 4 per condition. (**G**) Scheme of the mouse organoid culture experimental design. Representative immunofluorescence images of organoids from *Cpt1a*-floxed and *Cpt1a Spc*-KO mice (*n* = 3, per genotype) showing an increase in Scgb1a1 in *Cpt1a Spc*-KO mouse organoids. All scale bars: 50 μm.

To further characterize the intermediate states observed in challenged *Cpt1a Spc-*KO mice, we performed differential gene expression analysis. Remarkably, we found that among the top upregulated genes in KO mice was *Scgb1a1* (club secretory protein CC10, CCSP, or uteroglobin), a classical secretory lineage marker (35) (Figure 5A). *Scgb1a* expression was predominantly increased in the Intermediate 2 cell cluster and at a lower level in AT2, Intermediate 1, and AT1 cells of *Cpt1a Spc*-KO mice (Figure 5, A–C). Cells in the Intermediate 2 cell cluster in *Cpt1a Spc*-KO mice had low expression of AT2 (*Sftpc*, *Sftpb*) and ADI (*Krt8*, *Krt17*, and *Krt18*) cell markers (Figure 4C) and high expression of airway secretory cell markers, including *Scgb3a2*, *Cd24a*, *Muc5b*, and *Aldh1a1* (Figure 5C*)*. In contrast, the Intermediate 2 population in *Cpt1a*-floxed mice did not express markers of AT2, Intermediate 1, or ADI cells, but expressed high levels of *Sox2*, a critical regulator of airway cell identity known to prevent fate changes into alveolar cells (36) (Figure 5C). The Intermediate 2 cell population showed statistically enriched gene sets in pathways related to epithelial-mesenchymal transition (EMT), hypoxia, and cholesterol homeostasis (Figure 5D). This population also showed an increase in the expression of the genes of the glycolysis pathway (Supplemental Figure 5, A and B, and Supplemental Table 6). We performed immunofluorescence studies to validate the presence of Scgb1a1^+^ cells in lungs of *Cpt1a Spc*-KO mice. Our results revealed Scgb1a1^+^ cells in the peribronchial and interstitial areas of the injured lung in KO mice (Figure 5E). To confirm that deficiency of CPT1a promotes the onset of this population with a secretory phenotype, we analyzed PCLSs from ROSA^mT/mG^ SPC-Cre-ER mice with lineage tracing of AT2 cells (33, 34). PCLSs obtained after in vivo tamoxifen-induced recombination showed Scgb1a only in samples treated for 24 hours with the CPT1a inhibitor etomoxir (Figure 5F). Semiquantitative analysis indicated that the Scgb1a1^+^ cells were derived from AT2 cells, as they were also GFP^+^ (Figure 5F). In addition, we analyzed 3D organoids from EpCAM^+^ cells obtained from *Cpt1a*-floxed and *Cpt1a Spc*-KO lungs. Scgb1a1^+^ cells were detected by immunofluorescence predominantly in *Cpt1a Spc*-KO 3D cultures (Figure 5G). Taken together, these studies suggest that deficiency of CPT1a promotes a RAS phenotype in AT2 cells.

### Cpt1a loss increases the expression of senescence markers of cells in intermediate states.

**Figure 6:**
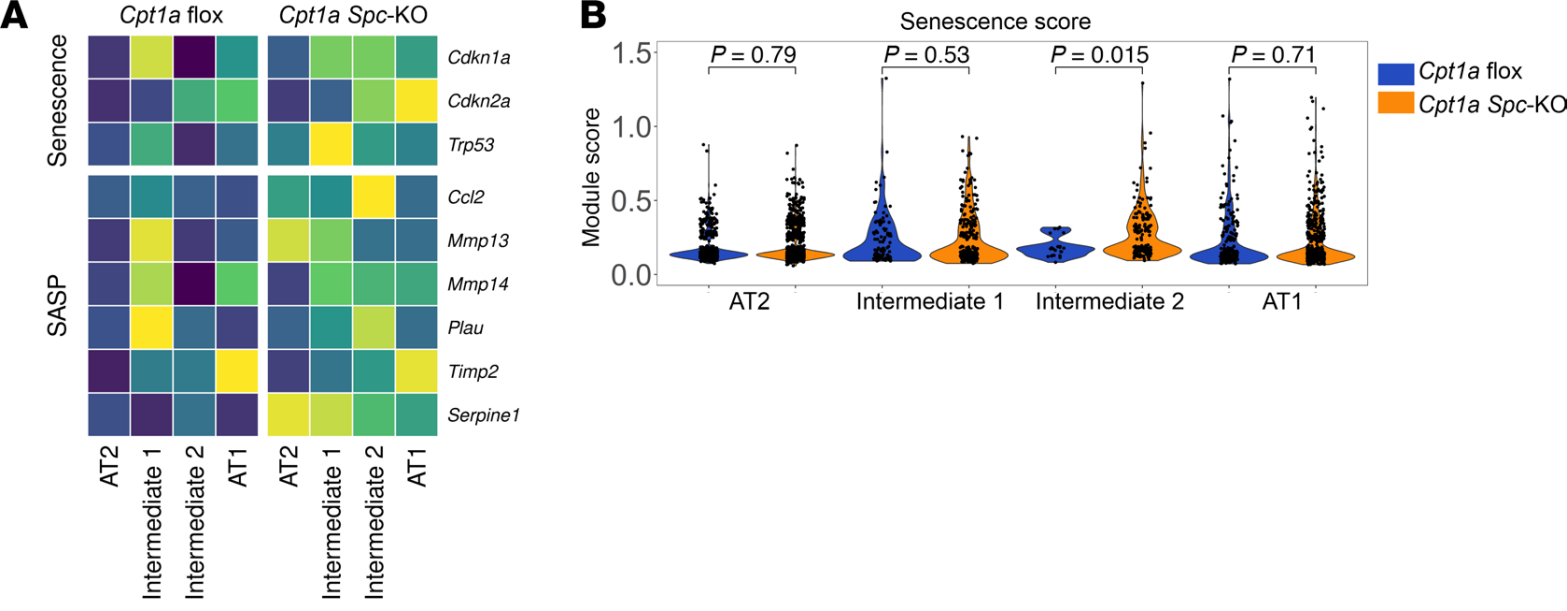
Loss of CPT1a promotes expression of senescence markers in lung epithelial cells. (**A**) Heatmap shows gene expression of senescence markers and senescence-associated secretory phenotype (SASP) genes in each epithelial cell population from MHV-68–infected *Cpt1a*-floxed (*n* = 4) and *Cpt1a Spc*-KO (*n* = 6) mice. (**B**) Senescence score in each epithelial cell population from MHV-68–infected *Cpt1a*-floxed (*n* = 4) and *Cpt1a Spc*-KO (*n* = 6) mice. Violin plots display the distribution of data; statistical significance was determined by Wilcoxon’s test.

Since ADI cells and pathological intermediate state cells featured in IPF lungs are characterized by increased senescence, we assessed the expression levels of senescence and senescence-associated secretory phenotype (SASP) gene markers in *Cpt1a-*floxed and *Cpt1a Spc-*KO animals. As expected, we found that the Intermediate 1 subcluster, which exhibited the previously described ADI phenotype, expressed markers of senescence in *Cpt1a-*floxed and *Cpt1a Spc-*KO animals (Figure 6A and Supplemental Table 7). However, it was also evident that all epithelial populations of the *Cpt1a Spc-*KO, including AT2, Intermediate 1, Intermediate 2, and AT1 cells had a higher expression level of genes associated with senescence and SASP compared with flox controls (Figure 6A and Supplemental Table 8). Calculation of a composite gene score confirmed that the senescence score for all epithelial cell populations was significantly higher in KO animals compared with *Cpt1a-*floxed (Figure 6B and Supplemental Tables 7 and 8), suggesting that loss of CPT1a leads to the establishment of a senescence phenotype.

**Figure 7:**
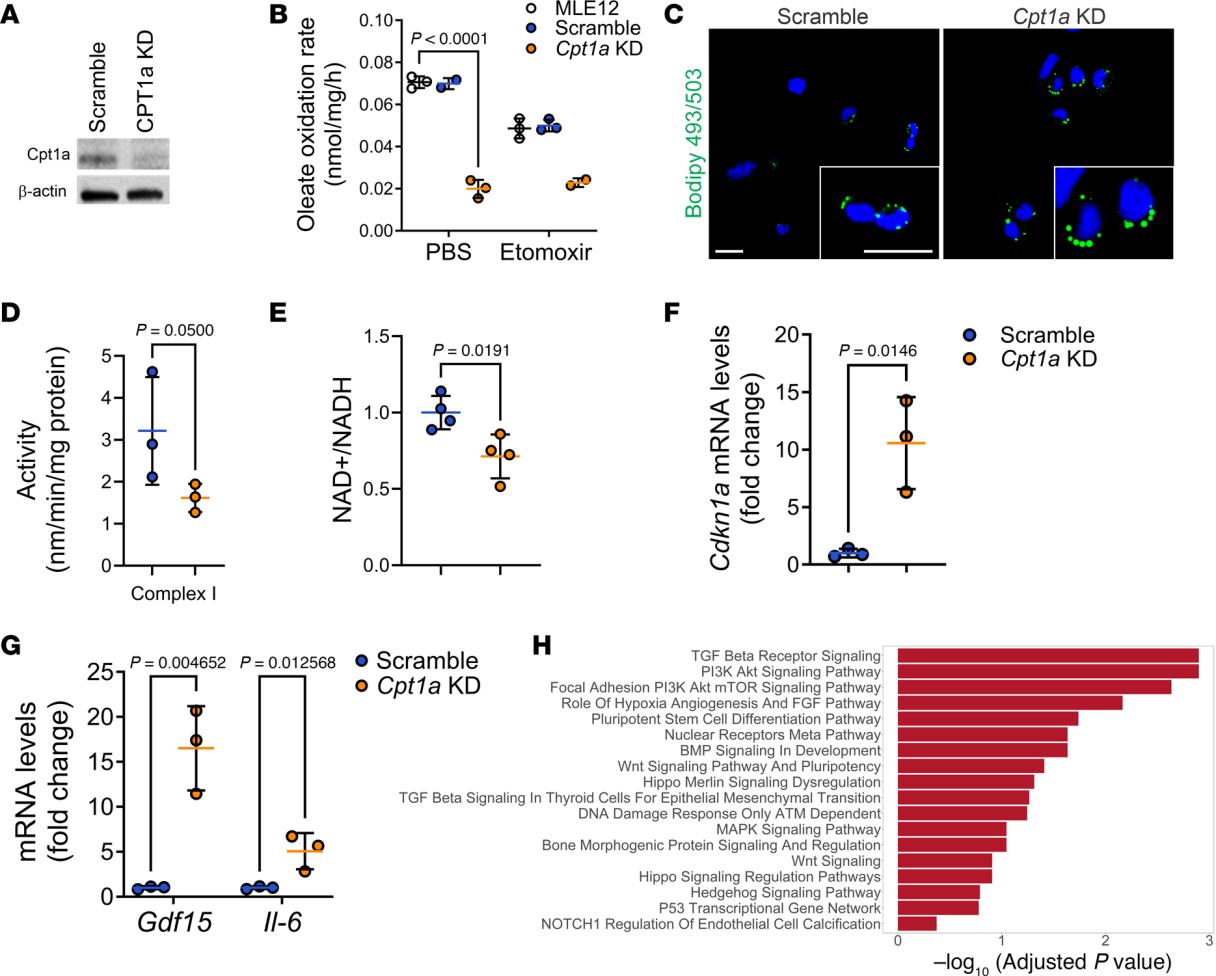
*Cpt1a* deficiency induces mitochondrial dysfunction. (**A**) Representative immunoblot showing efficient knockdown (KD) of *Cpt1a* in MLE 12 epithelial cells. (**B**) Oleate oxidation rate in *Cpt1a*-KD, scramble control, and parental MLE 12 cells (*n* = 3) in the presence and absence of CPT1a inhibitor etomoxir (MLE 12-PBS, *n* = 3; Scramble-PBS, *n* = 2; *Cpt1a* KD-PBS, *n* = 3; MLE12-PBS, *n* = 3; Scramble-PBS, *n* = 3; *Cpt1a* KD-PBS, *n* = 2). Data represent mean ± SD; statistical significance was determined by 2-way ANOVA followed by Tukey’s multiple-comparison test. (**C**) Representative images of BODIPY (lipid) staining of MLE 12 cells. Scale bars: 20 µm (**D**) Assessment of mitochondrial complex I activity in *Cpt1a*-KD compared with scramble MLE 12 cells (*n* = 3, per condition). Data represent mean ± SD; statistical significance was determined by Mann-Whitney *U* test. (**E**) NAD^+^/NADH ratio in *Cpt1a*-KD and scramble MLE 12 cells (*n* = 4, per condition). Data represent mean ± SD; statistical significance was determined by 2-tailed, unpaired Student’s *t* test. (**F**) Gene expression of senescence marker *Cdkn1a* in *Cpt1a*-KD and scramble (*n* = 4, per condition). Data represent mean ± SD; statistical significance was determined by Mann-Whitney *U* test. (**G**) mRNA levels of SASP genes (*Gdf15* and *Il6*) in scramble controls and *Cpt1a*-KD cells (*n* = 3, per condition). Data represent mean ± SD; statistical significance was determined by unpaired Student’s *t* test. (**H**) Enriched pathways analysis of differentially expressed genes in *Cpt1a*-KD versus scramble cells.

To further characterize the phenotype of lung epithelial cells with CPT1a deficiency, we used a mouse lung epithelial cell line (MLE 12) where *Cpt1a* expression was silenced using short hairpin RNA (shRNA) with a knockdown (KD) efficiency of approximately 80%, as assessed by Western blot (Figure 7A) and qPCR (Supplemental Figure 6A). We assessed FAO in *Cpt1a*-KD MLE 12 cells by measuring their ability to oxidize ^14^C-oleate (a long-chain FA that is >16 carbons). We found that MLE 12 cells treated with etomoxir, a CTP1a inhibitor, and *Cpt1a*-KD cells exhibited lower oxidative capacities than scramble controls or PBS-treated cells (Figure 7B). In agreement with impaired FAO, *Cpt1a*-KD cells also accumulated more lipid droplets than scramble control, as demonstrated by BODIPY 493/503 staining of neutral lipids (Figure 7C). Silencing *Cpt1a* also led to increased mitochondrial mass as demonstrated by MitoTracker staining (Supplemental Figure 6B), which was associated with higher expression of the mitochondria biogenesis regulator *Ppargc1a* (Supplemental Figure 6C). Further analysis showed that *Cpt1a*-KD cells exhibited decreased mitochondrial complex I activity (Figure 7D), concomitant with decreased NAD^+^ (Supplemental Figure 6D) and a lowered NAD^+^/NADH ratio (Figure 7E), key characteristics of a mitochondrial dysfunction–associated senescence phenotype (37). In concordance, we found increased expression of the senescence markers *Cdkn1a* (p21) and SASP genes *Gdf15* and *Il6* (Figure 7, F and G). RNA-seq data analysis confirmed top enriched pathways in *Cpt1a*-KD cells, similar to data obtained from the Intermediate 2 population, including hypoxia, mTOR, DNA repair, and differentiation pathways (TGF-β, BMP, Wnt, Hippo, Notch) (Figure 7H). Altogether, our data show that CPT1a deficiency leads to loss of metabolic homeostasis and impaired mitochondrial function, which in turn promotes activation of senescence and differentiation pathways.

### Loss of CPT1a induces TGF-β signaling activation.

**Figure 8:**
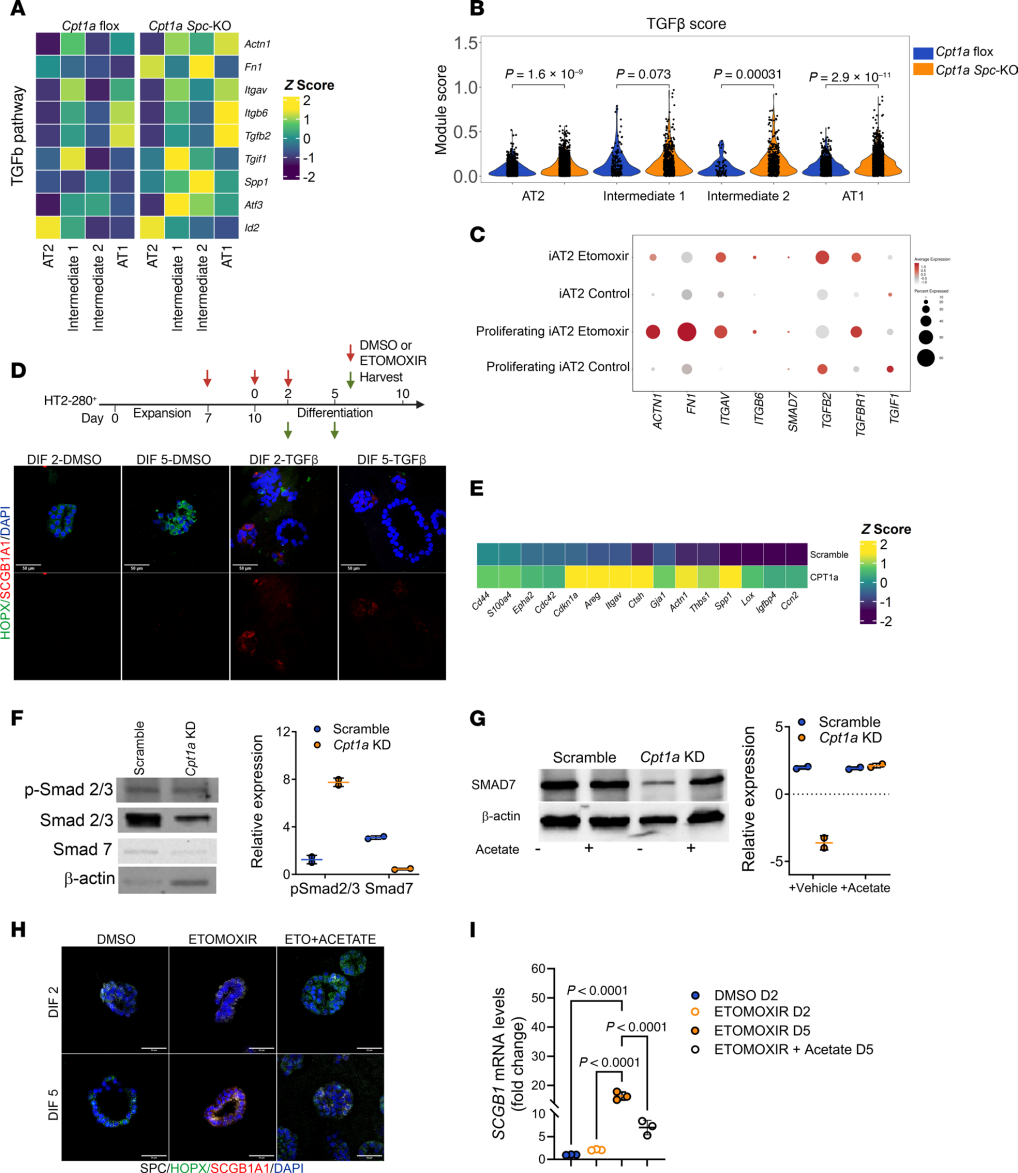
*Cpt1a* deficiency induces activation of the TGF-β pathway. Heatmap (**A**) and scores (**B**), showing the gene expression and activation of the TGF-β pathway in each epithelial cell population from MHV-68–infected *Cpt1a*-floxed (*n* = 4) and *Cpt1a Spc*-KO (*n* = 6) mice. Violin plots display the distribution of data, statistical significance was determined by Wilcoxon’s test. (**C**) Dot plot depicting gene expression of the TGF-β pathway in the cell populations from iAT2 organoids. (**D**) Top: Scheme of the human alveolar organoid culture experimental design in the presence of TGF-β treatment. Bottom: Representative immunofluorescence images showing the increased presence of the secretory cell marker SCGB1A1 (red) in organoids treated with TGF-β on day 2 or 5 of differentiation (*n* = 4, each group). HOPX (green) was used as an AT1 marker and KRT17 (white) was used as a transitional cell marker. Scale bars: 50 μm. (**E**) Heatmap shows the increased expression of TGF-β target genes in *Cpt1a*-KD cells compared with scramble (*n* = 3, per condition). (**F**) Representative Western blot images depicting levels of total Smad 2/3, Smad7, and p-Smad 2/3 on the left, with quantification on the right (*n* = 2, each group). Data represent mean ± SD. (**G**) Representative Western blot images depicting levels of Smad7 in *Cpt1a-*KD cells after acetate treatment on the left, with quantification on the right (*n* = 2, each group). Data represent mean ± SD. Representative immunofluorescence (**H**) and quantification (**I**) showing a decrease in SCGB1A1 expression in human organoids treated with etomoxir and acetate. Data represent mean ± SD. Statistical significance was determined by 1-way ANOVA followed by Tukey’s multiple-comparison test. Scale bars: 50 μm.

The TGF-β signaling pathway was found as a top enriched pathway in *Cpt1a*-KD cells and the Intermediate 2 cell population in *Cpt1a* *Spc*-KO mice. TGF-β signaling plays a critical role in AT2 differentiation, and an enrichment of TGF-β pathway genes has been reported in the ADI state, including in IPF lungs (38). To determine whether CPT1a deficiency modulates the TGF-β signaling pathway in alveolar epithelial cells, we analyzed canonical gene expression of TGF-β target genes in alveolar epithelial cell populations from the lungs of *Cpt1a Spc*-KO and *Cpt1*-flox mice using scRNA-seq. We then calculated a score to denotate TGF-β pathway activation (Supplemental Table 9). We observed that AT2, Intermediate 2, and AT1 cell populations from the *Cpt1a Spc*-KO mice have enhanced expression of TGF-β target genes in comparison with *Cpt1a*-floxed mice, confirmed by a composite gene score (Figure 8, A and B). In addition, expression of TGF-β target genes was increased in the iAT2 organoid culture system treated with etomoxir (Figure 8C and Supplemental Table 10). To confirm the role of TGF-β in our human AT2 cell differentiation model, we treated 3D human organoids with TGF-β. We found TGF-β treatment induced differentiation of AT2 cells into an intermediate cell phenotype expressing SCGB1A1 (Figure 8D). To further corroborate the effect of *Cpt1a* deficiency on TGF-β signaling, we used epithelial (MLE 12) *Cpt1a*-KD cells. Bulk RNA-seq and qPCR data demonstrated high expression of TGF-β pathway genes in *Cpt1a*-KD cells (Figure 8E, Supplemental Table 11, and Supplemental Figure 7A). In addition, *Cpt1a*-KD cells showed increased phosphorylation levels of TGF-β effectors SMAD2 and -3 in the absence of any stimuli compared with scramble controls, while levels of the TGF-β inhibitory factor SMAD7 were reduced (Figure 8F). One consequence of FAO inhibition is a lower availability to produce appropriate levels of acetyl-CoA that is utilized for acetylation of proteins. In concordance, we observed significantly lower acetyl-CoA levels in *Cpt1a*-KD cells compared with controls (Supplemental Figure 7B). Previous reports have shown that SMAD7 can be acetylated at its N-terminus by the lysine acetyltransferase p300, which prevents SMAD7 degradation via ubiquitination and therefore stabilizes the total levels of the SMAD7 protein (39). Since acetyl-CoA is the substrate for all acetylation reactions in cells, and its levels are decreased in *Cpt1a*-KD cells, we hypothesized that the lowered levels of total SMAD7 were due to its decreased acetylation, thereby affecting the stability of the protein. To test this hypothesis, we supplemented the growth media with acetate to restore cellular acetyl-CoA levels. Acetate supplementation was sufficient to restore SMAD7 protein in *Cpt1a*-deficient cells to similar levels observed in scramble controls (Figure 8G). Next, we asked whether acetate treatment could rescue the aberrant pattern of TGF-β target gene expression, specifically *Tgfb*, *Serpine1*, and *Fn1* (Supplemental Figure 7C). We observed that acetate treatment reduced the expression of transitional state cell markers Krt8 and Krt18 (Supplemental Figure 7D). Similarly, human lung epithelial organoids treated with etomoxir and supplemented with acetate significantly reduced protein and transcript levels of *SCGB1A1* and *KRT17* (Figure 8, H and I, and Supplemental Figure 7E). Collectively, these findings suggest that deficiency of CPT1A promotes the activation of the TGF-β pathway most likely through dysregulation of acetyl-CoA levels that contribute to the AT2 differentiation into intermediate cell states.

## Discussion

Lung tissue repair after injury is paramount to safeguarding tissue homeostasis and function. Efficient lung repair is achieved through the very well-coordinated process of alveolar regeneration. AT2 cells are the primary progenitor cells responsible for alveolar epithelial maintenance. Injury events promote AT2 cell expansion and differentiation into AT1 cells. The connection between altered AT2 cell differentiation and disease is highlighted by the persistent accumulation of cells in an AT2-AT1 intermediate state of differentiation expressing aberrant airway cell markers, which has been found in IPF patient lungs and in several murine models of lung fibrosis (20, 40). Alternative distinct populations of lung progenitor cells with the potential to differentiate into AT2 and AT1 cells that are derived from distal airways have also been described in mouse and human lungs (2, 7, 8, 41, 42). In the present study, we found that the FAO metabolic pathway plays a role in lung epithelial cell progenitor function. In addition, we found diminution of the FAO rate in aging AT2 cells and that defects in FAO alter the physiological alveolar regeneration after injury, leading to the accumulation of cells in an alveolar intermediate state with aberrant expression of basaloid and secretory airway phenotypes and higher susceptibility to developing lung fibrosis.

Cell differentiation generally requires well-orchestrated communication between signaling, transcriptional, and metabolic pathways. Supporting the key role of mitochondrial homeostasis in regulation of the fate of lung epithelial cells, recent studies demonstrate that mitochondrial complex I–NAD^+^ regeneration plays a role in alveolar epithelial cell fate during postnatal lung development (43). Deficiency of the complex I subunit NDUFS2 leads to accumulation of AT2-AT1 transitional cells associated with pathological activation of the integrated stress response. The FAO mitochondrial metabolic pathway has been shown to be involved in the regulation of cell fate in several physiological and pathological conditions, including cancer, EMT, kidney fibrosis, and immune cell activation (15, 16, 44). Importantly, the deletion of CPT1a in basal airway progenitor cells has been shown to impair the transition to full differentiation (19). Here, we show that deficiency of CPT1a in AT2 cells impairs FAO and mitochondrial respiration with diminution in complex I activity, NAD^+^ regeneration, and acetyl-CoA production.

In vitro and in vivo inhibition or deficiency of CPT1a in AT2 cells affected lung epithelial cell fate by promoting the accumulation of cells in intermediate states expressing AT2, AT1, and airway cell markers. These intermediate cells express elevated levels of cytokeratins, secretory cell markers, and genes associated with cell cycle arrest and senescence. Additionally, gene expression profiles and pathway enrichment analyses identified activation of TGF-β signaling pathway. Previous studies have shown that increased TGF-β signaling in endothelial cells is accompanied by decreased FAO, which is required to maintain endothelial acetyl-CoA levels (16). Our studies pinpoint impaired cellular pools of acetyl-CoA in lung epithelial cells leading to decreased levels of total Smad7, a negative regulator of the TGF-β pathway. Others have shown that acetylation of Smad7 at residues K64 and K70 can protect it from ubiquitin-mediated degradation (16, 39). This, together with our observation that acetate supplementation can rescue total levels of Smad7 to those observed in control cells, prompt us to propose that the persistent activation of the TGF-β pathway that is observed in FAO-deficient cells is most likely due to lack of acetylation. We cannot exclude the possibility that reduced acetylation might affect other signaling and transcriptional pathways as well as epigenetic transactions, which heavily rely on acetylation of histones that can be altered in FAO-deficient cells and can contribute to the phenotypes observed in this study. For instance, acetylation regulates the transcriptional function of *Sox2*, a key regulator of airway epithelial cell identity and plasticity (45). Importantly, TGF-β inhibition can replace Sox2 reprogramming and inhibition of TGF-β might be sufficient to reprogram trapped intermediate cells. Loss of Sox2 promotes KRT17^+^KRT5^–^ dysplastic epithelium after injury in the distal lung that is present in IPF lungs and patients with post-COVID-19 pulmonary fibrosis (36, 46).

FAO is also a source of many histone modifications (47). Changes in histone methylation have been shown to regulate the dynamics of distal lung epithelial progenitor cells (48). We show here that aging AT2 cells have low rates of FAO, implicating FAO deficiency in transcriptional regulation, chromatin remodeling, and epigenetic control of lung progenitor cell function. The mechanisms by which FAO regulates transcriptional programs that promote alveolar cell fate and AT2-AT1 differentiation are the focus of our future studies.

Epithelial populations that express both AT2 and airway epithelial cell markers, including cell markers of secretory or basal cells, can arise in the human lung during fibrotic processes (49). The merging of alveolar and secretory programs is characteristic of BASCs, which have been previously described in mice; this population has been shown to serve as progenitor cells in special circumstances, including severe lung injury and altered epigenetic regulation associated with aging (7, 9, 42, 48, 50). It has been demonstrated that the numbers of BASCs are relatively stable during lung homeostasis and that they rarely differentiate. However, BASCs could contribute to the generation of airway cells (club and ciliated cells) as well as AT2 and AT1 cells according to the type of lung injury (8). Specifically, after bleomycin-induced alveolar injury, BASCs contribute to fully differentiated AT2 and AT1 cells, but not club airway cells. Recent studies by 2 research groups have identified progenitor cells in human bronchioles that express alveolar and secretory lung cell markers. One group suggests that SCGB3A2 secretory cells undergo differentiation into AT2 cells (1). However, the second one describes a unique epithelial transition or intermediate state, termed the AT0 cell, originating from AT2 cells and expressing SFTPC, AGER, and SCGB3A2 (2). This AT0 cell can differentiate into either AT1 cells or terminal bronchiole secretory cells (SCGB3A2^+^/SFTPB^+^) when cultured in vitro. In cases of severe pulmonary fibrosis, these terminal bronchiole secretory cells form bronchiolized regions (2). Although our in vitro studies support the expression of secretory markers by AT2 intermediate cell states, further studies will be required to define the role of BASCs in the in vivo repair mechanism during lung injury in *Cpt1a* *Spc*-KO mice.

There are limitations to our studies. Although we have thoroughly demonstrated the critical role of CPT1A in AT2 progenitor cells, we have not fully uncovered the mechanisms by which FAO regulates AT2 differentiation. First, transcriptomic and epigenomic analysis will be needed to fully understand how CPT1a loss remodels the AT2 cells progenitor function. Second, although we have reported here enrichment of genes involved in glycolysis in lung epithelial cells deficient in CPT1a, metabolomic analysis will be needed to understand the compensatory mechanisms that these intermediate cells use to produce energy in the absence of FAO. Finally, studies to define whether pharmacological or genetic interventions that promote FAO are sufficient to improve lung repair and alveoli regeneration will be needed and might have important translational implications.

## Methods

Further information can be found in Supplemental Methods.

### Sex as a biological variable.

Our study examined male and female animals, and similar findings are reported for both sexes in this study.

### Statistics.

Statistical differences between 2 groups were compared using a Student’s *t* test. All other statistical analyses with more than 2 groups were determined by 1-way or 2-way ANOVA, followed by appropriate post hoc tests. *P* values of less than 0.05 were considered significant. Data are represented as individual dots ± SD of the mean and replicates are as indicated in figure legends. Statistical analyses were performed using GraphPad Prism software (version 10.1.2).

### Study approval.

Animal use was approved by the IACUC at the University of Pittsburgh and The Ohio State University and adhered to the NIH *Guide for the Care and Use of Laboratory Animals* (National Academies Press, 2011). Human lung tissues were collected from excess pathologic tissues after lung transplantation and organ donation, under The Ohio State University Institutional Review Board protocols 2017H0309, 2020H0512, and 2021H0180. All identifiers are managed through an Honest Broker Process detailed under approved IRB protocol 2017H0310. Demographics data from samples used in this study can be found in Supplemental Table 1.

### Data availability.

Further information and request for resources and reagents should be directed to and will be fulfilled by the corresponding author (Ana Mora: Ana.Mora@osumc.edu). All raw data can be found in the Supporting Data Values file. The datasets generated during this study are available at the NCBI Gene Expression Omnibus (GEO) under access numbers GSE283885 for human single-cell data, and GSE284440 and GSE284444 for fresh and fixed mouse single-cell data, respectively.

### Code availability.

The code used in this study is provided in GitHub (https://github.com/jhonny-rodriguez-lopez/Regulation_of_lung_progenitor_plasticity_and_repair_by_fatty_acid_oxidation).

## Author contributions

ALM conceived and designed the study. QDAL, JRL, PAG, JC, DA, MB, LNT, MST, NVA, JEK, AM, SB, SSS, VP, SS, MJ, MG, QM, TF, MR, and ALM contributed to experimental work, analysis, and interpretation. DA, JC, QDAL, BFR, PAG, and ALM drafted the manuscript and provided intellectual content. All authors approved the final version of the manuscript.

## Supplementary Material

Supplemental data

Unedited blot and gel images

Supporting data values

## Version 1 02/10/2025

Electronic publication
