# Supplementary material for: Regulation of lung progenitor plasticity and repair by fatty acid oxidation
Source: JCI Insight. 2025 Feb 10;10(3):e165837. doi: 10.1172/jci.insight.165837 (PMC11948574; doi:10.1172/jci.insight.165837)
Supplement: Supplemental data [file jciinsight-10-165837-s074.pdf]

Supplementary Table S1. Demographic characteristics of lung donor and patient cohorts

	**Old Donors**	**IPF**
**Single-Fixed RNA Profiling**		
Subjects	9	6
Age	64.3 ± 4.6 (58-72)	64 ± 5.8 (55-71)
Sex		
Female	5 (22.22%)	2 (33.33%)
Male	4 (77.78%)	4 (66.67%)

	**Young Donors**	**Old Donors**	**IPF**
**Immunofluorescence**			
Subjects	2	2	2
Age	18.5±0.7 (18-19)	69	70.5±2.1 (69-72)
Gender			
Female	1(50%)	1(50%)	1(50%)
Male	1(50%)	1(50%)	1(50%)
**Xenium**			
Subjects	2	2	2
Age	16.0±2.828 (14-18)	69 ± 4.243 (66-72)	57 ± 2.828 (55-59)
Sex			
Female		1(50%)	
Male	2(100%)	1(50%)	2(100%)
**Human alveolar organoids**			
Subjects	1		
Age	26		
Gender	Male		

Supplementary Table S2. Top 10 genes in each scRNAseq cluster (human data)

Cluster	Gene
AT2	*SFTPC*
AT2	*SFTPA1*
AT2	*LRRK2*
AT2	*PGC*
AT2	*CTSH*
AT2	*ABCA3*
AT2	*NAPSA*
AT2	*SFTPD*
AT2	*LAMP3*
AT2	*SFTPA2*
AT2 transitional	*CTSE*
AT2 transitional	*AQP4*
AT2 transitional	*GPRC5A*
AT2 transitional	*ICAM1*
AT2 transitional	*CEACAM6*
AT2 transitional	*HOPX*
AT2 transitional	*RNASE1*
AT2 transitional	*CLDN18*
AT2 transitional	*AQP1*
AT2 transitional	*SCGB3A2*
Basaloid	*CTSE*
Basaloid	*MMP7*
Basaloid	*TM4SF1*
Basaloid	*GSN*
Basaloid	*KRT7*
Basaloid	*KRT8*
Basaloid	*CEACAM6*
Basaloid	*S100A10*
Basaloid	*SCGB3A2*
Basaloid	*AQP1*
AT1	*AGER*
AT1	*RTKN2*
AT1	*EMP2*
AT1	*CAV1*
AT1	*VEGFA*
AT1	*GPRC5A*
AT1	*LMO7*
AT1	*SCEL*
AT1	*ANKRD29*
AT1	*CD55*

RAS	*SCGB3A2*
RAS	*KIAA1324*
RAS	*MMP7*
RAS	*CYB5A*
RAS	*SCGB3A1*
RAS	*WFDC2*
RAS	*RNASE1*
RAS	*PIGR*
RAS	*SLPI*
RAS	*MGP*
Proliferating Epithelial Cells	*HBD*
Proliferating Epithelial Cells	*SNCA*
Proliferating Epithelial Cells	*HBB*
Proliferating Epithelial Cells	*HBA2*
Proliferating Epithelial Cells	*FTL*
Proliferating Epithelial Cells	*VIM*
Proliferating Epithelial Cells	*SLC25A37*
Proliferating Epithelial Cells	*TMSB4X*
Proliferating Epithelial Cells	*IGKC*
Proliferating Epithelial Cells	*IGHG1*
Secretory cells	*BPIFB1*
Secretory cells	*CP*
Secretory cells	*SCGB3A1*
Secretory cells	*SCGB1A1*
Secretory cells	*MUC5B*
Secretory cells	*LCN2*
Secretory cells	*WFDC2*
Secretory cells	*PIGR*
Secretory cells	*SLPI*
Secretory cells	*SCGB3A2*
Goblet	*SCGB1A1*
Goblet	*CAPS*
Goblet	*C9orf24*
Goblet	*C20orf85*
Goblet	*FAM216B*
Goblet	*CLU*
Goblet	*TPPP3*
Goblet	*DDIT4*
Goblet	*SCGB3A1*
Goblet	*GLUL*
Basal	*KRT5*
Basal	*KRT15*
Basal	*KRT17*

Basal	*S100A2*
Basal	*COL7A1*
Basal	*TRIM29*
Basal	*SERPINF1*
Basal	*DDIT4*
Basal	*ZFP36L1*
Basal	*GLUL*
Ciliated	*CAPS*
Ciliated	*TPPP3*
Ciliated	*C9orf24*
Ciliated	*RSPH1*
Ciliated	*C20orf85*
Ciliated	*TSPAN1*
Ciliated	*CFAP157*
Ciliated	*FAM216B*
Ciliated	*ODF3B*
Ciliated	*MS4A8*

Supplementary Table S3. FAO target genes.

Gene Symbol	Description
CPT1A	Carnitine Palmitoyltransferase 1A
ACADL	Acyl-CoA Dehydrogenase, Long Chain
ACAA2	Acetyl-CoA Acyltransferase 2
ACAD10	Acyl-CoA Dehydrogenase Family Member 10
ACADM	Acyl-CoA Dehydrogenase, Medium Chain
ACADSB	Acyl-CoA Dehydrogenase, Short/Branched Chain
ACADVL	Acyl-CoA Dehydrogenase, Very Long Chain
ACOX1	Acyl-CoA Oxidase 1
ACOX2	Acyl-CoA Oxidase 2
ACSL1	Acyl-CoA Synthetase Long Chain Family Member 1
ACSL3	Acyl-CoA Synthetase Long Chain Family Member 3
ACSL4	Acyl-CoA Synthetase Long Chain Family Member 4
ADH5	Alcohol Dehydrogenase 5 (Class III), Chi Polypeptide
ALDH4A1	Aldehyde Dehydrogenase 4 Family Member A1
ALDH5A1	Aldehyde Dehydrogenase 5 Family Member A1
ALDH7A1	Aldehyde Dehydrogenase 7 Family Member A1
CPT2	Carnitine Palmitoyltransferase 2
HADH	Hydroxyacyl-CoA Dehydrogenase
HADHA	Hydroxyacyl-CoA Dehydrogenase Trifunctional Multienzyme Complex Subunit Alpha
HADHB	Hydroxyacyl-CoA Dehydrogenase Trifunctional Multienzyme Complex Subunit Beta
ACADS	Acyl-CoA Dehydrogenase, Short Chain
ACAT1	Acetyl-CoA Acetyltransferase 1
ACSL5	Acyl-CoA Synthetase Long Chain Family Member 5
ALDH1B1	Aldehyde Dehydrogenase 1 Family Member B1
EHHADH	Enoyl-CoA Hydratase And 3-Hydroxyacyl CoA Dehydrogenase
GCDH	Glutaryl-CoA Dehydrogenase

Supplementary Table S4. Gene markers for iAT2 organoids.

**Gene markers**	
Gene Symbol	Description
iAT2 cells	
SFTPC	Surfactant Protein C
SFTPB	Surfactant Protein B
NAPSA	Napsin A Aspartic Peptidase
NKX2-1	NK2 Homeobox 1
Proliferating iAT2 cells	
TOP2A	DNA Topoisomerase II Alpha
MKI67	Marker Of Proliferation Ki-67
iAT1 cells	
PDPN	Podoplanin
CAV1	Caveolin 1
VEGFA	Vascular Endothelial Growth Factor A

Supplementary Table S5. Top 10 genes in each cluster (mice data)

Cluster	Gene
AT2	*Sftpa1*
AT2	*Sftpb*
AT2	*Slc34a2*
AT2	*Cxcl15*
AT2	*Sftpc*
AT2	*Lyz2*
AT2	*Lcn2*
AT2	*Chil1*
AT2	*Scd1*
AT2	*Lamp3*
Intermediate 1	*Akap5*
Intermediate 1	*Anxa3*
Intermediate 1	*Vegfa*
Intermediate 1	*Mprip*
Intermediate 1	*Serpinb9*
Intermediate 1	*Ltbp3*
Intermediate 1	*Krt8*
Intermediate 1	*Clu*
Intermediate 1	*Thbs1*
Intermediate 1	*Gbp8*
Intermediate 2	*Scgb1a1*
Intermediate 2	*Tmsb4x*
Intermediate 2	*Cmss1*
Intermediate 2	*S100a6*
Intermediate 2	*Gm42418*
Intermediate 2	*Fth1*
Intermediate 2	*Apoe*
Intermediate 2	*Lgals3*
Intermediate 2	*Ctss*
Intermediate 2	*Cdk8*
AT1	*Spock2*
AT1	*Rtkn2*
AT1	*Ndnf*
AT1	*Vegfa*
AT1	*Ager*
AT1	*Pdpn*
AT1	*Ccn2*
AT1	*Pmp22*
AT1	*Col4a4*
AT1	*Gprc5a*

Supplementary Table S6. Glycolysis pathway genes (Supplementary Figure 5A, 5B).

**Glycolysis pathway**	
Gene Symbol	Description
Eno2	Enolase 2
Pfkl	Phosphofructokinase, Liver Type
Pgam2	Phosphoglycerate Mutase 2
Pkm	Pyruvate Kinase M1/2
Gpi1	Phosphatidylinositol Glycan Anchor Biosynthesis Class Q
Pfkm	Phosphofructokinase, Muscle
Eno1	Enolase 1
Pgk1	Phosphoglycerate Kinase 1
Hk1	Hexokinase 1
Eno3	Enolase 3
Pfkp	Phosphofructokinase, Platelet
Gapdh	Glyceraldehyde-3-Phosphate Dehydrogenase
Tpi1	Triosephosphate Isomerase 1

Supplementary Table S7. Senescence target genes (Supplementary Figure 6A, 6B).

Senescence target genes	
Gene Symbol	Description
Cdkn1a	Cyclin Dependent Kinase Inhibitor 1A
Cdkn2a	Cyclin Dependent Kinase Inhibitor 2A
Trp53	Tumor Protein P53

Supplementary Table S8. SASP target genes (Supplementary Figure 6A, 6B).

SASP target genes	
Gene Symbol	Description
Ccl2	C-C Motif Chemokine Ligand 2
Mmp13	Matrix Metallopeptidase 13
Mmp14	Matrix Metallopeptidase 14
Plau	Plasminogen Activator, Urokinase
Timp2	TIMP Metallopeptidase Inhibitor 2
Serpine1	Serpin Family E Member 1

Supplementary Table S9. TGF-β pathway genes (Supplementary Figure 8A, 8B).

**TGF-β pathway**	
Gene Symbol	Description
Actn1	Actinin Alpha 1
Fn1	Fibronectin 1
Itgav	Integrin Subunit Alpha V
Itgb6	Integrin Subunit Beta 6
Tgfb2	Transforming Growth Factor Beta 2
Tgif1	TGFB Induced Factor Homeobox 1
Spp1	Secreted Phosphoprotein 1
Atf3	Activating Transcription Factor 3
Id2	Inhibitor Of DNA Binding 2

Supplementary Table S10. TGF-β pathway genes (Supplementary Figure 8C).

**TGF-β pathway**	
Gene Symbol	Description
Actn1	Actinin Alpha 1
Fn1	Fibronectin 1
Itgav	Integrin Subunit Alpha V
Itgb6	Integrin Subunit Beta 6
SMAD7	SMAD Family Member 7
TGFB2	Transforming Growth Factor Beta 2
TGFBR1	Transforming Growth Factor Beta Receptor 1
TGIF1	TGFB Induced Factor Homeobox 1

Supplementary Table S11. TGF-β target genes (Supplementary Figure 8E).

**TGF-β target genes**	
Gene Symbol	Description
Cd44	CD44 Molecule (IN Blood Group)
S100a4	S100 Calcium Binding Protein A4
Epha2	EPH Receptor A2
Cdc42	Cell Division Cycle 42
Cdkn1a	Cyclin Dependent Kinase Inhibitor 1A
Areg	Amphiregulin
Itgav	Integrin Subunit Alpha V
Ctsh	Cathepsin H
Gja1	Gap Junction Protein Alpha 1
Actn1	Actinin Alpha 1
Thbs1	Thrombospondin 1
Spp1	Secreted Phosphoprotein 1

Lox	Lysyl Oxidase
Igfbp4	Insulin Like Growth Factor Binding Protein 4
Ccn2	Cellular Communication Network Factor 2

Supplemental Table S12. List of PrimeTime® primer assays (Integrated DNA Technologies)

Gene	Assay ID
SFTPC	Hs.PT.58.143199.g
KRT17	Hs.PT.58.20464487
LAMP3	Hs.PT.56a.20751516
AQP5	Hs00387048_m1
SCGB1A1	Hs.PT.58.1190800
KRT5	Hs.PT.58.14446018
SCGB3A2	Hs.PT.58.38916981
RN18S1	Hs.PT.47.122532.g
Cpt1a	Mm.PT.58.10147164
Rn18s	Mm.PT45.122532.g
Cdkn1a	Mm.PT.51.17125846
Cdkn2a	Mm.PT.51.5632963
Cdkn2d	Mm.PT.51.9881334
Gdf15	Mm.PT.58.13112185
Il-6	Mm.PT.49a.11799101.g
Ppargc1a	MmPT.49a.8439184
Fn1	Mm.PT.58.8135568
Col1a1	Mm.PT.47.12668954
Spp1	Mm.PT.58.43709208
Timp1	Mm.PT.53A.30682575
Tgfb1	Mm.PT.58.11254750
Serpine1	Mm.PT.58.6413525

Supplemental Table S13. List of SYBR primers

Primers		Sequence
Cpt1a	F<br>R	5’ – CTCAAACCTATTCGTCTTCTG – 3’<br>5’ – TTGGATGGTGTCTGTCCTC – 3’
ORF50	F<br>R	5’-GGCCGCAGACATTTAATGAC-3’<br>5’-GCCTCAACTTCTCTGGATATGCC-3’
Krt8	N/A	Mm.PT.58.6862465
Krt18	N/A	Mm.PT.58.1389639
Rn18s	F<br>R	5' ACGGACAGGATTGACAGATTG 3'<br>5' ATCGCTCCACCAACTAAGAAC 3'
GAPDH	F<br>R	5’- CCTGCACCACCAACTGCTTAG-3’<br>5’-GTGGATGCAGGGATGATGTTC-3’

Supplementary figures

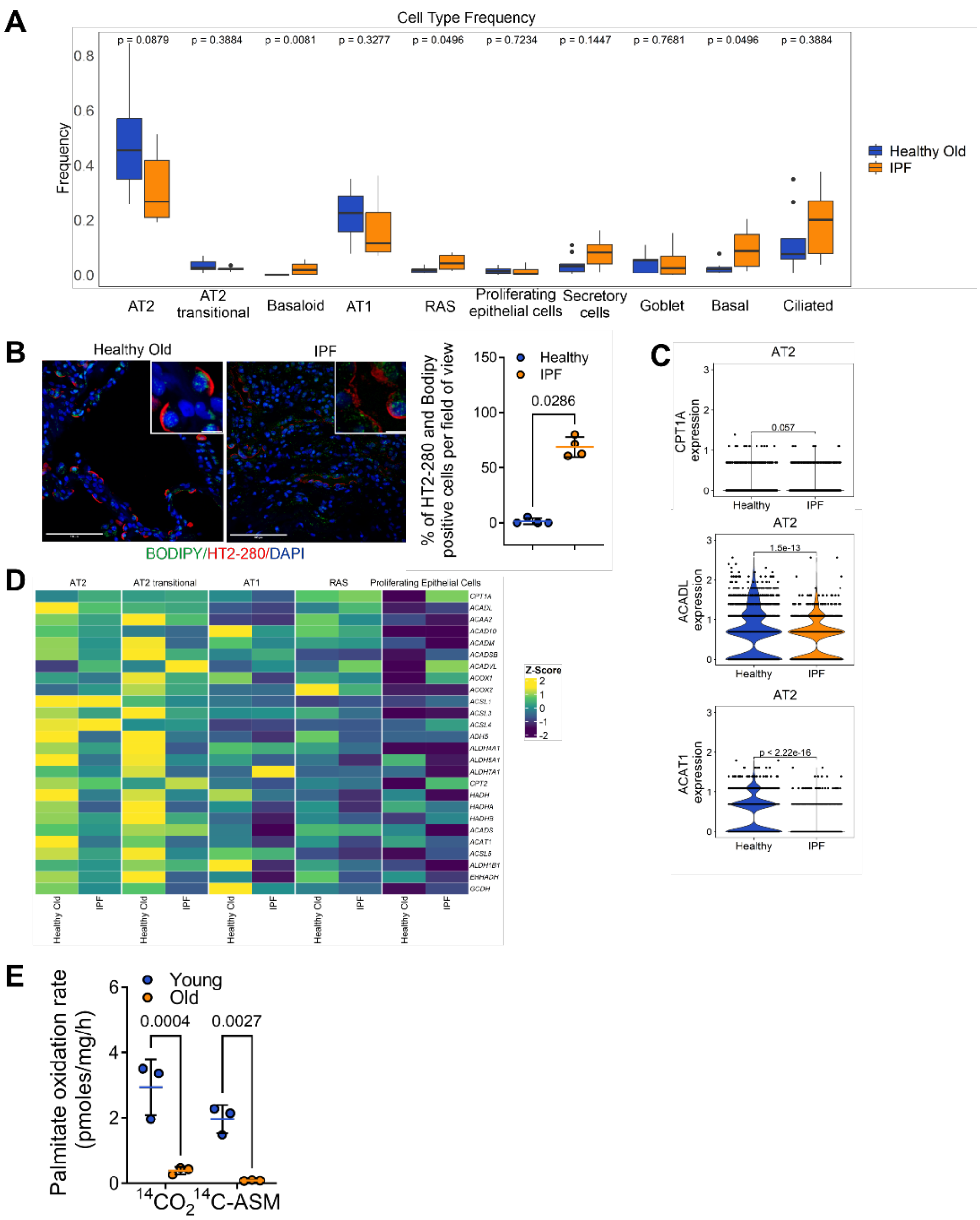

Supplementary Figure 1. (A) Whisker plots showing cell proportions of all epithelial and airway subtypes in healthy old donors (*n* = 9) and IPF (*n* = 6). Statistical significance was determined by Wilcoxon test. **(B)** Representative images and quantification of AT2 cells (HT2-280, red) and co-labeled lipid droplets (Bodipy; green) in healthy deceased donor and IPF lungs (*n* = 3, per group). Scale bar = 50 µm. Data represent mean ± SD; each dot represents a FOV. Statistical significance was determined by two-tailed Mann Whitney test. **(C)** Violin plots showing the expression of FAO enzymes in AT2 cells from healthy (*n* = 9) and IPF lungs (*n* = 6), statistical significance was determined by Wilcoxon test. **(D)** scRNA-seq heatmap of FAO gene expression in epithelial cells from healthy old deceased donor (*n* = 9), and IPF lungs (*n* = 6). Color scale denotes Z-score of the normalized fold change expression. **(E)** Rate of ^{14}C-palmitate oxidation in young and old mice (*n* = 3, per condition). Data represent mean ± SD; statistical significance was determined by two-way ANOVA followed Šídák's multiple comparisons test.

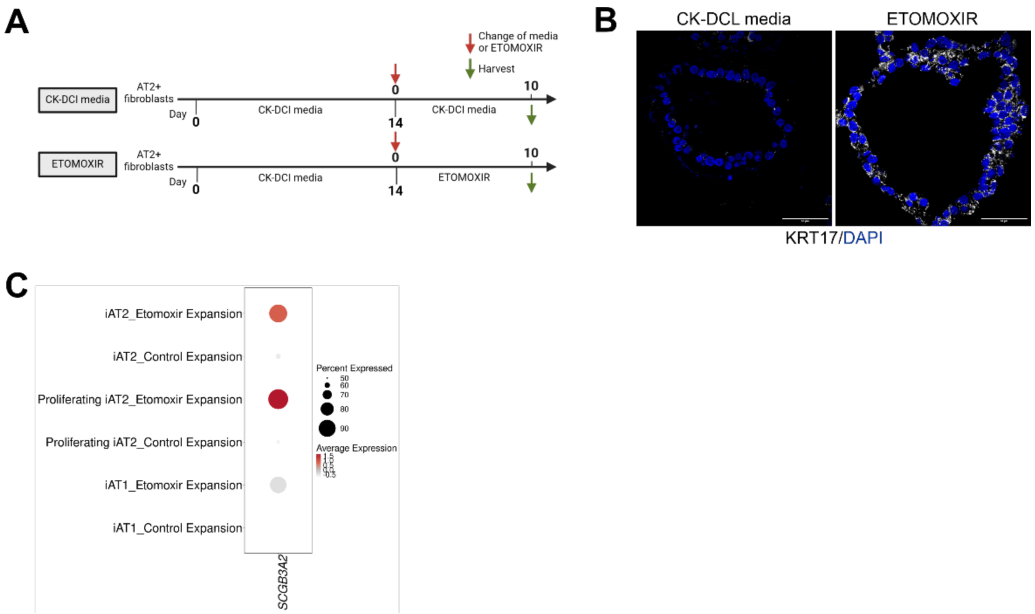

Supplementary Figure 2. (A) Timeline of the experiment. **(B)** Immunofluorescence depicting increased KRT17 expression in organoids from iAT2 cells treated with etomoxir. Scale bar = 50 µm. **(C)** Dot plot depicting the expression of *SCGB3A2* in the different cell populations treated and non-treated with etomoxir.

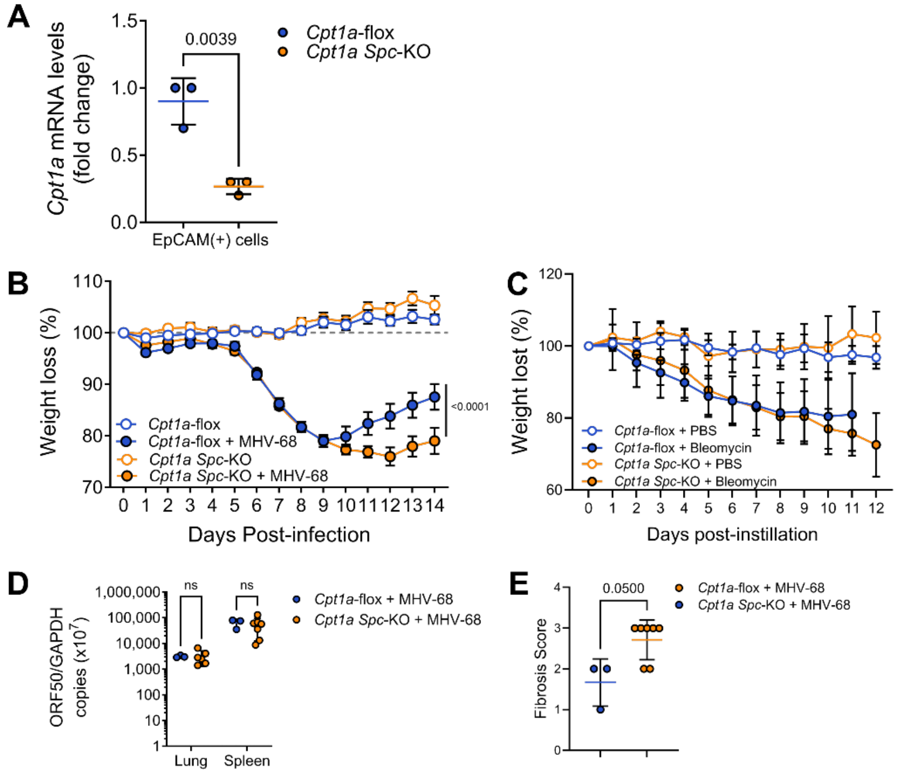

Supplementary Figure 3. (A) *Cpt1a* mRNA levels in EpCAM+ (epithelial) cells from floxed and *Cpt1a Spc*-KO mice (*n* = 3, per condition). Data represent mean ± SD; statistical significance was determined by two-tailed unpaired Student's t-test. **(B)** Weight loss curve of *Cpt1a*-flox and *Cpt1a Spc*-KO mice treated with bleomycin or PBS as a control (*Cpt1a*-flox + PBS *n* = 4, *Cpt1a*-flox + Bleomycin *n* = 9, *Cpt1a* Spc-KO + PBS *n* = 3, *Cpt1a Spc*-KO + Bleomycin *n* = 5). Data represent mean ± SD: statistical significance was determined by two-way ANOVA followed by Tukey's multiple comparison test. **(C)** Weight loss curve of *Cpt1a*-flox and *Cpt1a Spc*-KO mice, naïve or infected with the MHV-68 virus. Data represent mean ± SD. Statistical significance was determined by two-way ANOVA followed by Tukey's multiple comparison test (*Cpt1a*-flox *n* = 10, *Cpt1a*-flox + MHV-68 *n* = 14, *Cpt1a* Spc-KO *n* = 7, *Cpt1a Spc*-KO + MHV-68 *n* = 23). **(D)** ORF50 expression levels in lung and spleen from *Cpt1a*-flox (*n* = 3, per sample) and *Cpt1a Spc*-KO mice (lung, *n* = 6; spleen, *n* = 7) infected with the MHV-68 virus. Data represent mean ± SD; statistical significance was determined by two-way ANOVA followed Šídák's multiple comparisons test. **(E)** Fibrosis score in MHV-68-infected *Cpt1a*-flox (*n* = 3) and *Cpt1a-Spc*-KO mice (*n* = 7). Data represent mean ± SD; statistical significance was determined by and one-tailed unpaired Student's t-test.

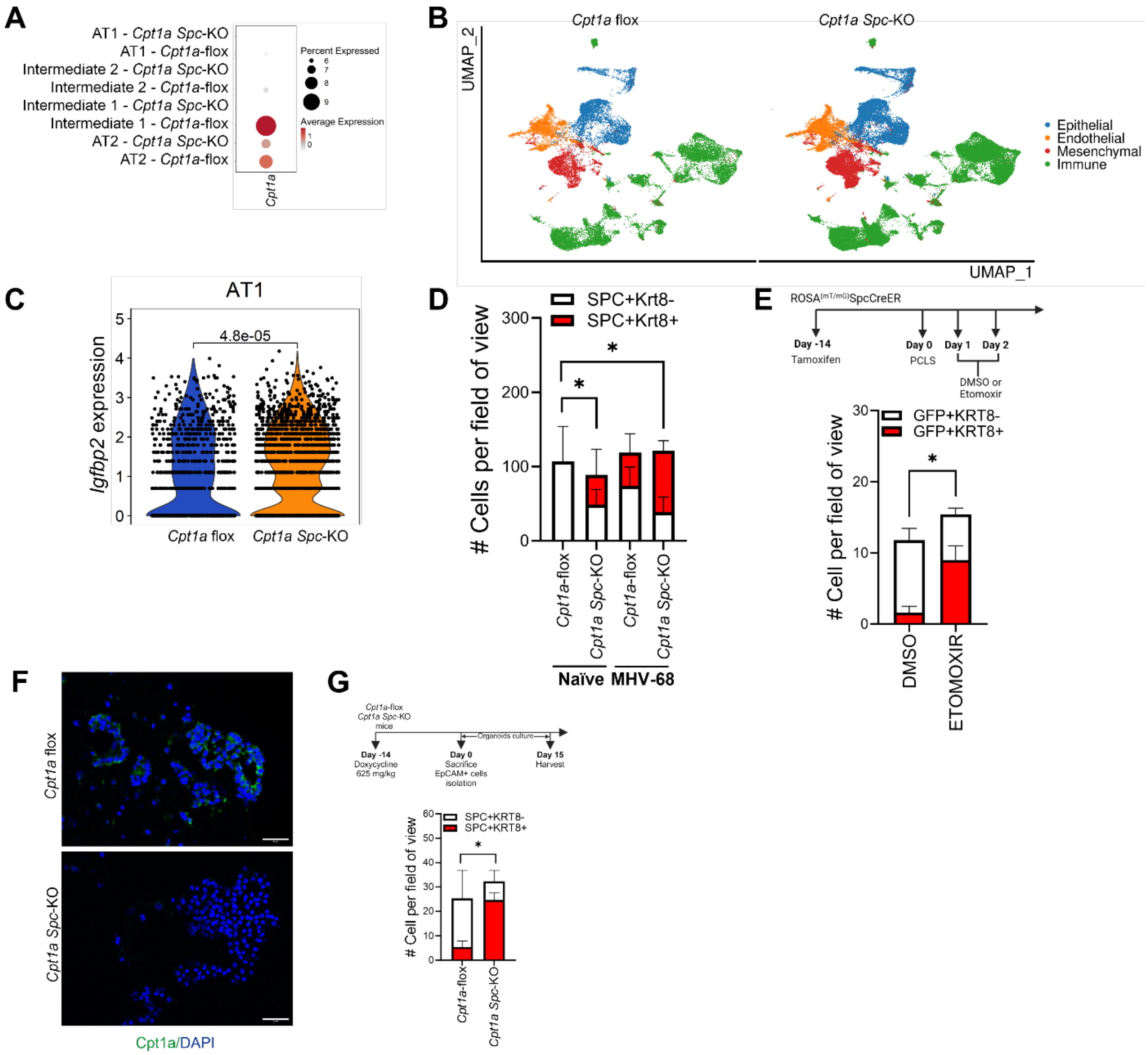

Supplementary Figure 4. (A) Dot plot showing the expression *Cpt1a* in epithelial cells from *Cpt1a*-flox and *Cpt1a Spc*-KO mice. **(B)** UMAP showing the distribution of the main four lineages in *Cpt1a*-flox and *Cpt1a Spc*-KO mice. **(C)** Violin plot showing the expression of *Igfbp2* in AT1 cells from *Cpt1a*-flox and *Cpt1a Spc*-KO mice. **(D)** Quantification of Krt8+ cells in naïve or MHV-68-infected *Cpt1a* floxed and *Cpt1a Spc*-KO mice. Data represent mean ± SD: statistical significance was determined by two-way ANOVA followed by Tukey's multiple comparison test (*Cpt1a*-flox $n = 5$, *Cpt1a*-flox + MHV-68 $n = 3$, *Cpt1a* Spc-KO $n = 5$, *Cpt1a Spc*-KO + MHV-68 $n = 4$), *p<0.05. **(E)** Top, scheme of the experiment. Bottom, quantification of cells GFP+Krt8+ and GFP+Krt8- in precision cut lung slices (PCLS) from ROSA$^{(mT/mG)}$ SPCCreER mice treated with vehicle or Cpt1a inhibitor. Data represent mean ± SD: statistical significance was determined by two-way ANOVA followed by Bonferroni's multiple comparison test, $n = 5$ per condition, *p<0.05. **(F)** Immunofluorescence depicting decreased Cpt1a expression in *Cpt1a Spc*-KO organoids compared to flox controls. **(G)** Top: scheme of the mouse organoid culture experimental set up, bottom: quantification of Spc+Krt8+ and Spc+Krt8- cells in immunofluorescence images of organoids from *Cpt1a* floxed and *Cpt1a Spc*-KO mice ($n = 3$, each group). Data represent mean ± SD: statistical significance was determined by two-way ANOVA followed by Bonferroni's multiple comparison test, *p<0.05. Scale bar = 50 μm.

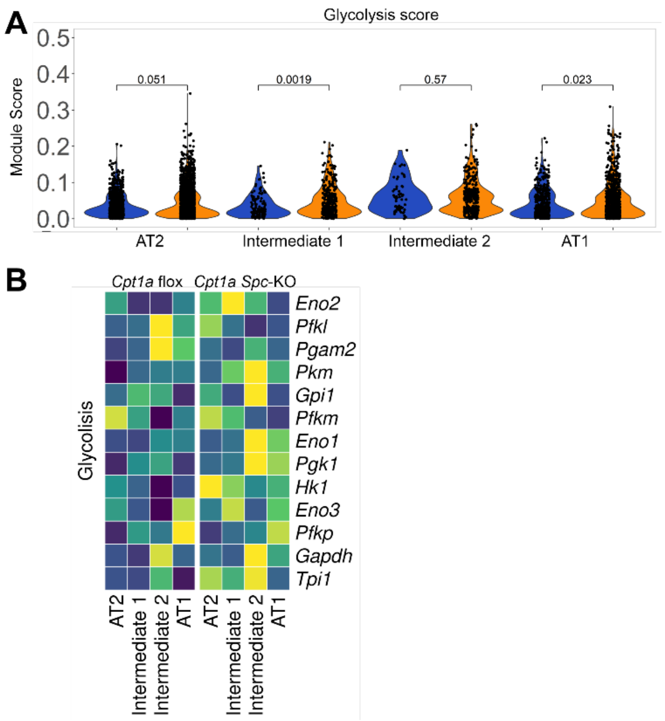

Supplementary Figure 5. Glycolysis score **(A)** and heatmap **(B)** in each epithelial cell population from floxed- (n = 6) and *Cpt1a Spc*-KO- (n = 8) infected mice. Violin plots display the distribution of data, statistical significance was determined by Wilcoxon test.

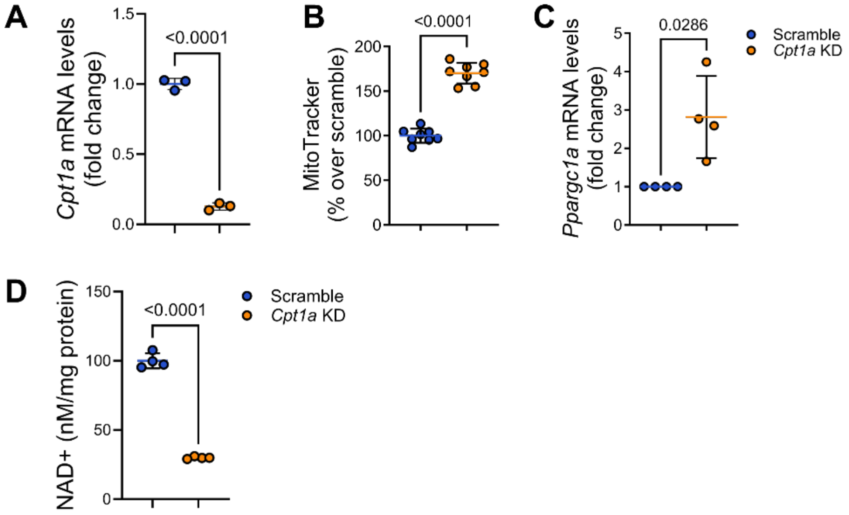

Supplementary Figure 6. (A) qPCR assessing gene expression levels of *Cpt1a* in *Cpt1a* KD vs. scramble MLE 12 cells (n = 3, per group). **(B)** Mitochondrial mass assessment as measured by MitoTracker staining of *Cpt1a* KD compared to scramble cells (n = 8, per group). Data represent mean ± SD; statistical significance was determined by two-tailed unpaired Student's t-test. **(C)** Mitochondria biogenesis regulator *Ppargc1a* mRNA expression in *Cpt1a* KD and scramble MLE 12 cells (n = 4, per condition). Data represent mean ± SD; statistical significance was determined by Mann-Whitney U test. **(D)** NAD^+ mass assessment in *Cpt1a* KD and Scramble control (n = 4, per group). Data represent mean ± SD; statistical significance was determined by two-tailed unpaired Student's t-test.

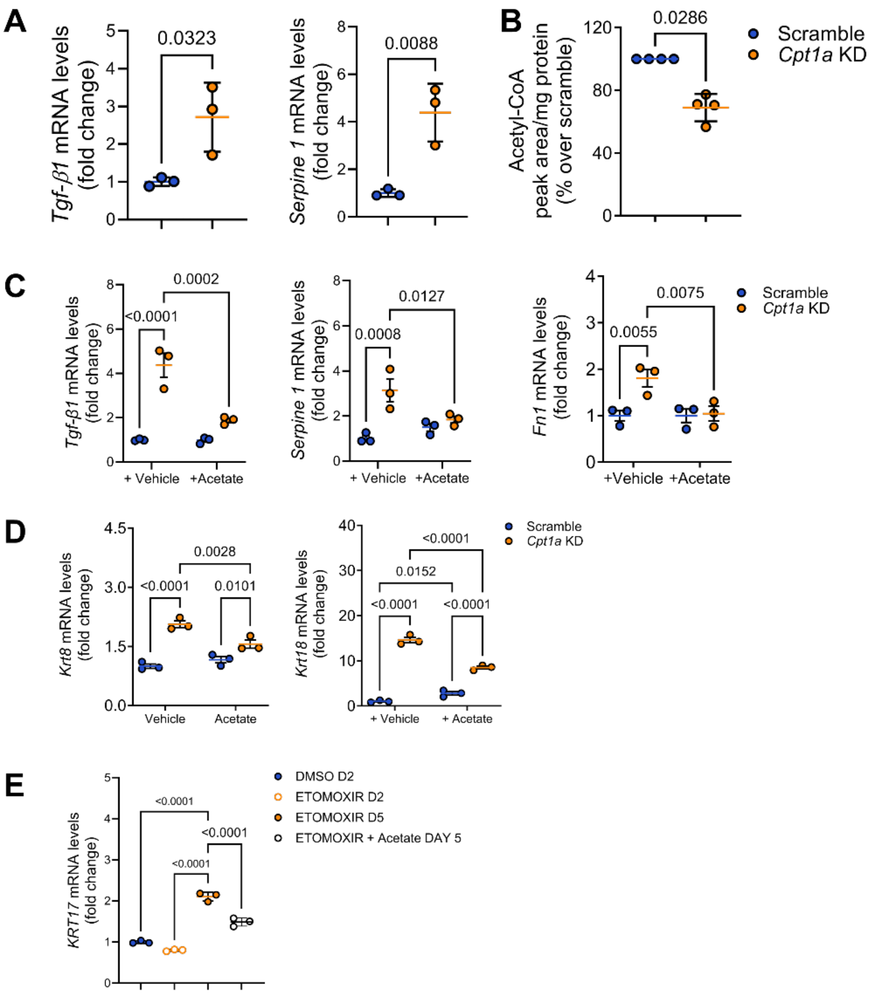

Supplementary Figure 7. (A) mRNA levels of *Tgf-β1* and *Serpine1* in scramble (*n* = 3) and *Cpt1a* KD (*n* = 3) cells. Data represent mean ± SD; statistical significance was determined by two-tailed unpaired Student's t test. **(B)** Acetyl-CoA quantification in scramble and *Cpt1a* KD MLE 12 cells (*n* = 4, per condition). Data represent mean ± SD; statistical significance was determined by Mann-Whitney *U* test. **(C)** mRNA expression levels of *Tgf-β1*, *Serpine 1* and *Fn1* in scramble controls and *Cpt1a* KD cells upon acetate treatment or vehicle control (*n* = 3, per condition). Data represent mean ± SD; statistical significance was determined by two-way ANOVA followed by uncorrected Fisher's LSD. **(D)** mRNA expression levels of *Krt8* and *Krt18* in scramble controls and *Cpt1a* KD cells upon acetate treatment or vehicle control (*n* = 3, per condition). Data represent mean ± SD; statistical significance was determined by two-way ANOVA followed by uncorrected Fisher's LSD. **(E)** *KRT17* expression in human AT2 organoids treated with DMSO, etomoxir and etomoxir + acetate. Data represent mean ± SD: statistical significance was determined by one-way ANOVA followed by Tukey's multiple comparison test.

Supplementary materials and methods

Sex as a biological variable*:*

Our study examined male and female animals, and similar findings are reported for both sexes in this study.

Generation of *Cpt1a Spc*-KO mice

Mice were housed in an approved USDA OLAW-registered and AAALAC-accredited facility at the University of Pittsburgh in Optimice© cages in ventilated racks on a 12hr light/12hr dark cycle with access to a standard chow diet at a density of 4 males or 5 females per cage. Young (2–6 months) C57BL/6J mice were acquired from Jackson Laboratories. Conditional type II lung epithelial cell *Cpt1a* knockout mice (*Cpt1a/tetO-cre/SFTC-rtTA*) were generated based on the tetO-cre/SFTC-rtTA previously described (1). The *Cpt1a flox/flox* (sites flanking exon 4) mice were obtained from Dr. Toren Finkel and are available at Jackson Laboratories (032778). All mice were on a C57BL/6J background. For the detection of the floxed *Cpt1a* by PCR genotyping, the primers used were: Fwd (5'- AGTATGTTTGAGAGACTGGCAAAGC-3') and Rev (5'-GTGCAGCAGAGTGGCCTGTCTCT-3'). Amplicon sizes are 250bp (*Cpt1a* WT) and 400bp (*Cpt1a* fl/fl). For TetO-cre, the primers used were: Tg-fwd (5'-GCG GTC TGG CAG TAA AAA CTA TC-3'), Tg-rev (5'-GTG AAA CAG CAT TGC TGT CAC TT-3'), WT-fwd (5'-CTA GGC CAC AGA ATT GAA AGA TCT-3'), and WT-rev (5'-GTA GGT GGA AAT TCT AGC ATC ATC C-3'). Amplicon sizes are 324bp (internal control) and 100bp (Tg). For SFTPC-rtTA primers used: Tg-fwd (5'-CAA ATG TTG CTT GTC TGG TG-3'), Tg-rev (5'-GTC AGT CGA GTG CAC AGT TT-3'), WT-fwd (5'-CGC TGT GGG GCA TTT TAC

TTT AG-3’) and WT-rev (5’-CAT GTC CAG ATC GAA ATC GTC-3’). Amplicon sizes are 200bp (internal control) and 450bp (Tg).

Two weeks before injury, homozygous *Cpt1a Spc*-KO were doxycycline-induced (625 mg/kg in chow, ENVIGO). Lung fibrosis was induced by oropharyngeal administration of a single dose of 0.75U/kg bleomycin as previously described (2), and lungs were harvested 12 days after injury. Lung fibrosis by viral infection was induced by intranasal inoculation of $5x10^4$ PFU of MHV-68 as previously described (3, 4), and lungs were harvested 9- or 14-days post-infection.

Generation of *Spc-CreER-ROSA$^{(mTmG)}$* mice

Sftpc$^{tm1(cre/ERT2)Blh}$/J (Spc-CreER) and ROSA$^{(mTmG)}$ mice were acquired from Jackson Laboratories (028054 and 007676, respectively) and backcrossed to a C57BL/6J background for more than 2 generations. Spc-CreER-ROSA$^{(mTmG)}$ mice were administered tamoxifen (20 mg/kg i.p.; Sigma, C8267) in corn oil (Sigma, T5648), and, after one week, were used for experiments. For the detection of Spc-CreER mice, the primers used were: common (5’-TGC TTC ACA GGG TCG GTA G-3’), Tg-Rev (5’-ACA CCG GCC TTA TTC CAA G-3’) and WT-Rev (5’-CAT TAC CTG GGG TAG GAC CA-3’). Amplicon sizes are 327bp (internal control) and 210bp (Tg). For the detection of ROSA$^{mT/mG}$ the primers used were: WT-Fwd (5’-AGG GAG CTG CAG TGG AGT AG-3’), common (5’-CTT TAA GCC TGC CCA GAA GA-3’) and Tg-Fwd (5’- TAG AGC TTG CGG AAC CCT TC-3’). Amplicon sizes are 212bp (WT) and 128bp (Tg).

PCLS from mice lungs

After two weeks of the tamoxifen administration (20 mg/kg i.p.; Sigma, C8267) in corn oil (Sigma, T5648), Spc-CreER-ROSA$^{(mTmG)}$ mice were euthanized, the lungs were perfused with 1 mL of PBS through the right ventricle. Then, a catheter was inserted into the trachea, the trachea was tied to the catheter with silk sutures to avoid leakage, and lungs were inflated with 1% low melting agarose (Invitrogen, 16520-100) dissolved in PBS. Lungs were immediately covered with ice to solidify the agarose. Lungs were then dissected and submerged in ice-cold PBS. To create the precision-cut lung slices (PCLS), the lungs were sliced at a 400 µm thickness using a vibratome (Leica, VT 1200S) and culture in 24 well plates in DMEM media supplemented with FBS 10% and antibiotics/antimycotics 1%; the media was change every hour in the first four hours of the culture. After 24 hours in culture, the media of the PCLS were either supplemented with DMSO or etomoxir (3mM) and keep for 24 hours in culture. Then, PCLS were wash with PBS and fixed with PFA 4% in PBS for 10 minutes at RT and prepared for the paraffine embedding.

Isolation of primary type 2 alveolar epithelial cells

Human AT2 cells were obtained as previously described (5). Isolation of mouse AT2 cells was performed as described (3).

Human alveolar organoids culture

After human AT2 cells were isolated, cells were resuspended in 1 mL of cold complete PneumaCult AvOE Medium (StemCell Technologies, 100-0847). A cell count was performed and the cell suspension was diluted to a concentration of $1.6x10^5$ cells/mL

using the same media. Matrigel (Corning, 354230) was then added in a 1:1 concentration, and 50 µL of the mix was placed in the center of a 24-well plate (StemCell Technologies, 100-0097) to create a dome/droplet shape. After 40 minutes at 37°C, PneumaCult AvOE Seeding Medium was added and the culture was maintained in a 37°C incubator for 2 days. On day 2 (expansion day 0), a full-media change was performed by adding complete PneumaCult AvOE Medium, and the media was changed every 3 days until expansion day 7 when the different treatments were added to the media. At day 10 of expansion, the differentiation of human alveolar organoids was started, and the media was changed to PneumaCult Alveolar Organoid Differentiation Medium (StemCell Technologies, 100-0861). Samples were collected at day 10 of expansion, days 3, 5 and 10 of differentiation depending of the experiment.

Mouse alveolar organoids culture

Once we collected AT2 cells from mice lungs, we resuspended the cells in 3D culture media and seeded the alveolar mouse organoids as described (6).

Culture of iPSC

The BU3 NGST iPSC line utilized in this study were provided by Prof. Darrell N. Kotton, Center for Regenerative Medicine, Boston University, Boston, MA, USA. BU3 NGST iPSC line was propagated on Matrigel in mTesR medium, with medium changes every day. Undifferentiated iPSCs were passaged onto fresh Matrigel every 4–5 days.

Differentiation of iPSC to lung progenitor cells and alveolar type 2 (iAT2) cells

The iPSCs were differentiated to lung progenitor cells and type 2 cells in a directed differentiation protocol (7, 8). STEMdiff Definitive Endoderm Kit (Stem Cell Technologies) was used to induce definitive endoderm following the manufacturer's protocol. After approximately 72 to 84 h, cells were harvested and analyzed by flow cytometry to check the efficiency of definitive endoderm induction by looking at the CD117 and CXCR4 double-positive cell markers (8, 9).

After definitive endoderm induction, cells were dissociated into small clumps and passaged at 1:6 on matrigel-coated plates in serum-free differentiation media (SFDM) (IMDM/F12 (1:3) (Life technologies), N2 (Life Technologies), B27, 50 µg/ml ascorbic acid, 2 mM Glutamax, 0.4 µM monothioglycerol, 0.05% BSA) supplemented with 2 µM dorsomorphin (Sigma-Aldrich) and 10 µM SB431542 (Tocris) for 72 h. Y-27632 (10 µM, Tocris) was added for the first 24 h only (9).

For induction of early-stage lung progenitor cells, anterior foregut endoderm was cultured in SFDM supplemented with 3 µM CHIR99021 (Tocris), 10 ng/ml rhBMP4 (R&D Systems), and 50–100 nM Retinoic acid (Sigma-Aldrich). Medium change was done every other day for 8-9 days (9).

To generate type 2 (iAT2) alveolospheres, on day 15 of differentiation progenitor cells were dissociated with 0.05% trypsin (Thermo Fisher Scientific) and sorted for NKX21+ cells. Sorted cells were suspended in growth factor reduced Matrigel at 400 cells/µl density. 50 µl Matrigel droplets were seeded in 6 well plates and incubated at 370C for

30 min. Once Matrigel solidified, 2-3 ml differentiation medium containing SFDM supplemented with 3 μM CHIR99021, and 10 ng/ml rhKGF, 50 nM dexamethasone (Sigma-Aldrich), 0.1 mM 8-bromoadenosine 3′,5′-cyclic monophosphate (8-Br-cAMP) sodium salt (Sigma-Aldrich), and 0.1 mM 3-isobutyl-1-methylxanthine (IBMX) (Sigma-Aldrich), henceforth called as alveolosphere media, was added to the plate. Y-27632 (10 μM) was added to the medium for the first 48 h. Medium change was done every 48 h for two weeks for alveolospheres formation (7).

Co-culture of fibroblast and iAT2 cells

Fibroblast from healthy donors were used for co-culture with iAT2 cells in 3D organoid culture models. For co-culture with fibroblasts, alveolospheres were dissociated into a single-cell suspension. iAT2 cells and fibroblasts were co-cultured at a 1:2 ratio in the 3D system. iAT2 and fibroblast cells mixture were thoroughly mixed in 100% growth factor reduced matrigel (Corning, 354234) in ice. 50 μl droplets of Matrigel containing 5×104 cells were dispensed in the 6-well plate and were incubated at 370C for 30 min. Once Matrigel solidified, 2-3 ml of CKDCI medium was added to each well. Medium change was done every other day for 14 days for fibroblasts-iAT2 alveolospheres formation. After two weeks for the alveolosphere cultures were treated with Etomoxir two weeks and were collected for imaging or dissociated into single cells for sc-RNA-seq (10).

Histopathology, immunofluorescence, and electron microscopy

After sacrifice, lungs were perfused with 2% paraformaldehyde, followed by paraffin embedding. Histopathologic changes and fibrosis were evaluated using Masson's

Trichrome staining. Tissue immunofluorescence was performed using antibodies against CPT1a (Proteintech, 15184-1-AP), SPC (Santa Cruz Biotechnology, sc-7706), PDPN (R&D Systems, AF3244), Krt8 (Millipore, MABT329), GFP (Invitrogen, MA5-15256), HOPX (Santa Cruz Biotechnology, sc-3987703), AGER (R&D Systems, MAB1179-100), KRT17 (Abcam, ab53707), SCGB1A1 (R&D Systems, MAB4218) Lamp3 (Synaptic Systems, 391 005) and HT2-280 (Terrace Biotech, TB-27AHT2-280). Semiquantitative analyses of cells double-positive for HT2-280 and other proteins were performed in ImageJ.

For lipid accumulation, after the secondary antibody incubation, the slides were incubated for 1 hr with 20 µm/mL BODIPY 493/503 (Invitrogen, D3922) at room temperature in the dark.

For electron microscopy, tissues were fixed in 3% glutaraldehyde and 2% paraformaldehyde in a 0.1 M sodium cacodylate buffer at pH 7.3. Tissues were then dehydrated and embedded in pure epon. Ultrathin sections were examined using JEOL JM-1011 electron microscope.

Radiolabeled fatty acid oxidation assay

Fatty acid oxidation assay was performed as previously described (11). ^{14}C-palmitate was diluted with unlabeled palmitate and conjugated to fatty acid-free BSA in serum/glucose-free DMEM. Reactions contained 1 µCi/mL of radioactivity, corresponding to a 125 µM final concentration of palmitate. Freshly isolated mouse lung AT2 cells were resuspended in serum/glucose-free DMEM supplemented with 5 mM glucose and 200 µM free carnitine. Reactions were started by adding the ^{14}C-palmitate-BSA and were incubated

at 37°C. One hour post incubation, 0.5mM perchloric acid was added and released $^{14}CO_2$ was captured on filter papers soaked in 1M KOH. One hour after capture, filter papers were removed and subjected to scintillation counting. The reaction tubes were cooled on ice for 1 hr, centrifuged, and the supernatant was extracted with methanol/chloroform as previously described (12). Extracted acid-soluble metabolites (ASM) were quantified. The radioactive counts from captured $^{14}CO_2$ and extracted ^{14}C-ASM were normalized to total cellular protein and divided by the specific activity of the ^{14}C-palmitate-BSA to yield rates of FAO in pmoles of palmitate oxidized per µg per hr.

Cells

MLE 12 cells (ATCC, CRL-2110) were grown in HITES media (ATCC) supplemented with 2% FBS (Gibco), 50 U/mL penicillin, and 50 µg/mL streptomycin (Gibco), and incubated at 37°C in 5% CO_2. For gene knockdown, cells were transduced using scramble (CCTAAGGTTAAGTCGCCCTCG) and shCPT1a (ATGGACTCTAGTGATACAAAC) lentiviral supernatants with 8 µg/mL polybrene (Sigma) for 24 hrs. Four days after transduction, positive cells were selected in puromycin at 1 µg/mL for 48 hrs. Then, half a dose of puromycin was kept daily as maintenance.

For lipid accumulation, live cells were stained with 1 µM BODIPY 493/503 (Invitrogen, D3922) and 1 µM Hoechst (Molecular Probes) at 37°C for 30 mins in the dark.

Oleate oxidation rate assay

Oxidation of oleate to carbon dioxide was performed as previously described (11) with minor modifications. The day of the experiment, media was changed to plain DMEM

containing 0.7% fatty acid-free BSA (Sigma, A9205), 250 μM unlabeled oleate (Sigma, O3880), 1 μCi/mL-1 [1-14C] oleate (PerkinElmer, NEC317050UC), 1 mM carnitine (Sigma, C0283), 11 mg/L phenol red (Sigma, P3532) and 1.5 g/L sodium bicarbonate (Sigma, 792519) and incubated at 37°C. After 3 hours, $^{14}CO_2$-containing media was moved to glass tube (Thermo Fisher Scientific, 14-961-26) with an inner microdialysis tube (Thermo Fisher Scientific, 02-681-229) holding 1 M NaOH (Fisher Scientific, S3201) to capture CO_2. The tube was sealed and 1 M percholoric acid (Sigma, 244252) was injected into collection tube. After capturing overnight, NaOH-containing $^{14}CO_2$ was moved to scintillation tubes containing scintillation fluid (PerkinElmer, 6013119). Radioactivity was determined by counts per minute using an LS 6500 Multi-Purpose Scintillation Counter (Beckman Coulter).

Analysis of mitochondrial bioenergetics

Oxygen consumption rate (OCR) was measured by the Seahorse XF96 Extracellular Flux analyzer (Seahorse Bioscience) and was performed as previously described (3) with minor modifications. Primary AT2 cells isolated from CPT1a KO and WT mice were seeded in a Cell-Tak pre-coated plate in complete alveolar epithelial cell medium (ScienCell) and with etomoxir (40 μM; Cayman Chemical) and/or Palmitate-BSA (Seahorse Bioscience). After 1 hr of incubation, 2 μM oligomycin, 2 μM FCCP (carbonyl cyanide-p-trifluoromethoxyphenylhydrazone), and 2 μM rotenone were sequentially added; changes in OCR were measured in real time every 3-5 mins. Data were normalized per crystal violet.

Measurement of acetyl-CoA

Metabolic quenching and polar metabolite pool extraction was performed using ice cold 80% methanol/0.1% formic acid at a ratio of 500 μL/10^6 cells. 17:0 heptadecanoyl Coenzyme A (Avanti Polar Lipids Inc) was added to samples as an internal standard. After 3 mins of vortexing, the sample was centrifuged, and 2 μL of cleared supernatant was subjected to untargeted liquid chromatography-high resolution mass spectrometry (LC-HRMS). Samples were injected via a Thermo Vanquish UHPLC and separated over a reversed-phase Phenomenex Luna C18 (2) column maintained at 55°C. For the 20 min LC gradient, the mobile phase consisted of solvent A (water/5mM ammonium acetate) and solvent B (CAN/5mM ammonium acetate). Thermo IDX tribrid mass spectrometer was operated in positive ion mode, scanning data dependent MS^2 mode (2 μscans, top 10) from 150 to 1500 m/z at 70,000 resolution with an AGC target of 2e5. Source ionization setting was 3.0 kV spray voltage. Source gas parameters were 35 sheath gas, 12 auxiliary gas at 320°C, and 8 sweep gas. Calibration was performed prior to analysis using the PierceTM FlexMix Ion Calibration Solutions. Integrated peak areas were then extracted manually using Quan Browser (Thermo Fisher Xcalibur ver. 2.7). Three different sets were run, and data were normalized to protein content.

qPCR

Tissue and cell total RNA from was extracted using an RNeasy kit (Qiagen). qPCR was performed as previously described (3). Relative gene expression was obtained using $2^{-\Delta\Delta Ct}$ method under housekeeping normalization (r18s RNA). Premixed primers and probes (assay number in Supplemental Table S12) were from Integrated DNA Technologies.

SYBR primers and their sequences (Supplemental Table S13) were validated and obtained from PrimerBank database.

Western blot

Immunoblots were performed as described (3). The primary antibodies used were: phospho-SMAD2(Ser465/467)/SMAD3(Ser423/425) (Cell Signaling, 8828), anti-SMAD2/3 (Cell Signaling, 8685), CPT1A (Abcam, 15184-1-AP), SMAD7 (Abcam, AB216428) and anti-β-actin (Proteintech, 66009-1-Ig).

Human lung tissue preparation

Human lung tissues were collected from excess pathologic tissues after lung transplantation and organ donation, under The Ohio State University Institutional Review Board Protocols **2017H0309, 2020H0512**, and **2021H0180**. All identifiers are managed through an Honest Broker Process detailed under approved IRB Protocol **2017H0310**. Tissue was collected by the OSU Comprehensive Transplant Center (CTC) Human Tissue Biorepository, adhering to NCI and ISBER Best Practices for Repositories, from the parenchymal region of the lower lobes of explanted lungs from patients diagnosed with IPF and from age-matched healthy donor lungs. A demographic summary of the study population is provided in Supplementary Table S1. Pieces of the lung tissue (10 mm X 20 mm) were fixed for 24 h in 4% PFA and transferred to 70% ethanol and embedded on paraffin to have a Formalin-Fixed Paraffin-Embedded (FFPE) lung tissue block.

Data can be accessed at the Gene Expression Omnibus.

Mouse lung tissue preparation for FFPE analysis

Murine whole lung tissue was harvested, fixed in 10% neutral-buffered formalin, transferred to 70% ethanol, and paraffin-embedded.

FFPE RNA isolation and quality control

RNA isolation was performed on FFPE human (healthy donor, n = 9 and IPF, n = 6) and murine whole lung tissue (MHV-68-infected *Cpt1a* flox (n = 3) and *Cpt1a Spc*-KO (n = 5) mice). Two to three 10 μm scrolls were cut from each FFPE sample using a microtome (Leica, HistoCore BIOCUT) for isolation of total RNA using RNeasy DSP FFPE Kit (Qiagen, Cat No. 73604). ≥30% DV200 metric was used to determine the RNA input requirement and effectively parse samples suitable for NGS from unsuitable samples.

Fixed RNA Profiling from FFPE

Two 50-μm scrolls from each FFPE sample were used as input for dissociation into a single-cell suspension according to Demonstrated Protocol CG000632 using 1 mg/ml Liberase TH, Rev B by 10x Genomics. The fixed RNA profiling was performed based on human or mouse probes to identify whole transcriptome and conducted with the Chromium X platform, with a target of 128,000 cells for capture, 8,000 cells per sample for a single reaction of 16 samples, each sample with a barcode. For library construction, DNA was amplified after 10 PCR cycles for indexing (Dual Index Plate TS Set A, Chromium Fixed RNA Profiling Reagent Kits for Multiplexed Samples, CG000527, Rev F, 10x Genomics).

Next-generation sequencing was carried out in the Advanced Genomics Core at the University of Michigan and in the Novogene Corporation Inc.

Gene expression libraries were sequenced on Illumina NovaSeq 6000 sequencer with sequencing depth of 15,000 read pairs per cell, Paired-end, dual indexing type, read lengths of 28 cycles Read 1, 10 cycles i7 index, 10 cycles i5 index, 90 cycles Read 2. scRNA-seq data was extracted from the raw sequencing data using Cell Ranger (version 7.1.0, 10x Genomics).

Single-cell RNA sequencing analysis in humans

Raw sequencing reads were processed using the Cell Ranger pipeline (v7.1.0, 10x Genomics) for quality assessment and alignment to the human reference genome. The reference datasets used were *refdata-gex-GRCh38-2020-A* and *Chromium_Human_Transcriptome_Probe_Set_v1.0.1_GRCh38-2020-A* provided by 10x Genomics to generate a unique molecular identifier count matrix that was used to create a Seurat object containing a count matrix and analysis. Seurat object was combined into a merged dataset, and we removed the ambient RNA a percentage of mitochondrial genes, hemoglobin genes, and ribosomal genes were calculated for each sample in the merged object. The processed data for each study was imported using R 4.2.3 and Seurat v4.4.0. Samples were integrated using the Seurat and Harmony packages in R were normalized using the *SCTransform* function with default parameters and scale the data, and dimensionality reduction was performed using PCA on the top 3000 most variable genes. To correct batch effects and integrate the datasets, the

IntegrateData function was applied. For clustering, the *FindNeighbors* and *FindClusters* functions were used.

EPCAM+ and CDH1+ were used as canonical gene markers for epithelial cells. Each cell subtype was split into clusters and manually annotated with known cell type markers. "*SFTPC*", "*HHIP*", "*ABCA3*","*LAMP3*", "*PGC*", “*SFTPA2*","*SFTPB*","*SERPINA1*" and "*NAPSA*" were used to identify AT2 cells. “*ITGB6*”,"*ANXA1*", "*KRT8*", "*KRT18*", "*CLDN4*", "*SFN*", and "*KRT7*" were used to identify AT2 transitional cells “*MMP7*”,”*CDKN1A*” and “*GDF15*, were used to identify Basaloid cells. "*PDPN*", "*CAV1*", "*CLIC5*", "*AGER*", "*RTKN2*", "*HOPX*", "*GPRC5A*", "*EMP2*", "*CLDN18*", and "*CLIC3*" were used to identify AT1 cells. “*SFTPB*” and"*SCGB3A2*", were used to identify RAS cells. “MKI67” and “*CDK1*” were used to identify Proliferating Epithelial Cells. "*TP63*","*CDH3*", "*S100A2*", "*KRT5*", and "*KRT17*" were used to identify Basal cells. "*TPPP3*", "*FOXJ1*", "*TMEM190*", "*CAPS*", and "*HYDIN*" were used to identify Ciliated cells. "*MUC5B*", "*SCGB1A1*", “*SCGB3A2*”,”*SCGB3A1*” and "*BPIFB1*" were used to identify Secretory cells. “*MUC5AC*” was used to identify Goblet cells.

We evaluated the expression of fatty acid metabolism in healthy donors and IPF patients at the single-cell RNA-seq level using data from healthy old and healthy young donors and IPF patients. FAO-related gene expression in epithelial cells was evaluated and z-values for each gene were visualized using heatmaps and barplots. The FAO genes were obtained from previous reports (13).

The expression of TGF-β pathway-related genes in epithelial cells was evaluated, and z-values for each gene were visualized using heatmaps and bar plots. The TGF-β pathway genes were obtained from previous studies (14).

RNA seq data for the analysis of mouse samples from fresh isolated samples

RNA-Seq libraries were prepared with the TruSeq Stranded Total RNA kit (Illumina, SanDiego, CA) following manufacturer's protocol. Mitochondrial and rRNA were depleted from total RNA by Ribo-Zero Plus followed by RNA fragmentation and cDNA synthesis. Blunt ended cDNA fragments were A-tailed followed by ligation of indexed sequencing adapters. PCR amplification allowed enrichment of DNA with adapters ligated to both ends and was followed by library quantity and quality control by Qubit and Agilent DNA 1000 TapeStation. Final libraries were normalized to 10nM pooled and diluted. Flowcells for NextSeq 500 were loaded with 1.6pM denatured library for automated cluster formation and 2x75 paired end sequencing, approximately 60 million reads per sample. Reverse stranded paired-end RNA-Seq reads, generated by Illumina Stranded Total RNA Ribo-Zero Plus kit, were checked for presence of adapters and high-quality bases using FastQC(v 0.11.9). Universal adapters in the high-quality reads were already trimmed, and therefore did not require the use of an adapter trimming tool. Reads were later mapped in Ensemble mouse reference genome (GRCm38 mm10) using HISAT2(v 2.2.0) mapping tool. The output file was converted from SAM to BAM format using SAMtools(v 1.10).

Single-cell RNA sequencing analysis of *Cpt1a Spc*-KO mouse lungs

Raw sequencing reads underwent quality assessment and alignment to the mouse reference genome GRCm38 using the Cell Ranger pipeline v7.1.0 (10X Genomics) (15). All downstream bioinformatics analyses were conducted using R 4.2.3 and Seurat v4.4.0. Cells exceeding 10% mitochondrial content and detecting fewer than 200 genes, 20%

ribosomal genes, and 5% hemoglobin genes were filtered out. Subsequently, individual samples were normalized with the negative binomial model SCTransform (16), followed by merging Seurat objects. To mitigate batch effects, we employed the anchor-based method in Seurat (17), considering 3000 highly variable genes. The anchors for each sample were determined using the FindIntegrationAnchors function, and integration was performed with IntegrateData. Principal components (PCs) were calculated using the RunPCA function.

Data clustering was then performed using the Louvain algorithm in R with a resolution parameter of 0.6 and 30 PCs. Subsequently, cells were projected using the UMAP algorithm. Canonical marker genes were employed to identify major cellular lineages, including epithelial (*Epcam*+), endothelial (*Pecam1*+), mesenchymal (*Col1a1*+), and immune (*Ptprc*+) populations. The same clustering workflow was applied specifically to the epithelial cells. We calculated the most expressed genes in each cluster generated using FindAllMarkers, considering only clusters related to alveolar epithelial cells based on gene expression and cell proximity in the UMAP. The names of the clusters were delineated using previously documented marker genes (14, 18).

Differentially expressed genes between conditions were detected using the FindMarkers function with a Wilcox test for each alveolar epithelial cell cluster, and the results were projected onto a jitter plot. In addition, to evaluate specific signatures, we used the AverageExpression function to determine the expression of genes associated with senescence, SASP, and TGF-β. Z-values were computed for each set across different alveolar cells by condition. The transcriptomic signatures were visualized using ComplexHeatmap v3.18 (19) in R. Furthermore, we calculated the combined z-value

scores for each set of genes across different conditions and represented them using bar plots.

RNA-Sequencing, and Bioinformatics analysis for iAT2 organoids

To generate single cells from alveolospheres for sc-RNA sequencing first alveolospheres were released from the Matrigel by incubating droplets in dispase for 1 h, centrifuged at 300 g, for 1 min, washed in PBS. Single-cell suspensions were generated by incubating alveolospheres in 0.05% trypsin for 30 min at 37C, washed in PBS, passed through a 40 mm filter, counted, and resuspended.

After cell density was determined by cell counter, 16000 cells per sample were loaded onto Chromium Single Cell Controller (10X Genomics) and libraries were prepared with Chromium Single Cell 3' Reagent Kits V3 chemistry (10X Genomics) following manufacturer’s recommendation. The size distribution and concentration of libraries were determined by High Sensitivity D1000 ScreenTape Assay (Agilent) and KAPA Library Quantification Kits (Roche). All libraries were normalized to 1.5nM, pooled together evenly and sequenced on an Illumina NovaSeq 6000 sequencer using NovaSeq 6000 S1 Reagent Kit (100 cycles) with 10X Genomics’ recommended read-length settings.

Organoid samples subjected to control and Etomoxir treatments were processed and analyzed using the Seurat package in R. Unique molecular identifier (UMI) counts were normalized and scaled using SCTransform, which applies regularized negative binomial regression to account for technical variation. Cells were filtered based on stringent quality control criteria, including mitochondrial content below 15%, total RNA features between 200 and 7,000, and the exclusion of low-quality cells. For downstream analyses,

individual datasets were merged into a unified Seurat object, with unique cell identifiers added to preserve sample traceability. Data normalization and scaling were performed using SCTransform, which accounts for technical variation through regularized negative binomial regression. To integrate datasets and identify shared biological features, we selected 3,000 integration features using the SelectIntegrationFeatures function and prepared datasets with PrepSCTIntegration. Integration anchors were computed with the FindIntegrationAnchors function, and an integrated dataset was generated using IntegrateData. Dimensionality reduction was performed using principal component analysis (PCA), with the top 10 principal components used to construct a Uniform Manifold Approximation and Projection (UMAP) for visualization. Cell clusters were identified using the FindNeighbors and FindClusters functions with a resolution parameter of 0.2. UMAP plots were generated to visualize the transcriptional landscape. We performed an unsupervised clustering analysis to group cells into distinct clusters based on transcriptomic similarity. The expressions of iAT2 markers, such as "*SFTPC*", "*SFTPB*" and "*NAPSA*", were used for annotation. "*TOP2A*" and "*MKI67*" were used to identify Proliferating iAT2 cells.

Downstream Analysis and Visualization

Jitter plots

To identify which genes were changed, differential gene expression analysis was performed using the FindMarkers function of the Seurat package. This analysis identified genes that were differentially expressed between *Cpt1a*flox MHV-68 and *Cpt1a Spc*-KO

MHV-68 in various epithelial cell types, such as AT2, Intermediate 1, Intermediate 2, and AT1.

Jitter plots showing the expression (y axis) of differentially expressed marker genes in each cell type (x axis). Significant genes were highlighted with a distinct color and labeled with their corresponding names. A dashed horizontal line at a log2 fold change value of 0.5 was included to emphasize genes with notable expression changes. Each dot in the jitter plot shows the expression of the genes in each cell type (Figure 5A).

Pathway analysis

To gain a more comprehensive characterization of the pathways altered upon *Cpt1a* loss, we performed bulk RNA-seq analysis on *Cpt1a* KD and scramble control cells. We identified a total of 805 differentially expressed genes (DEGs) using a cutoff of FDR (0.05) and log2 fold change values (≥ 1 or ≤ -1). Gene Ontology analysis of these DEGs revealed that the most enriched pathways were TGF-beta receptor signaling, PI3K-Akt signaling, and focal adhesion PI3K-Akt-mTOR signaling using the WikiPathways database through the online platform enrichR (Figure 7H).

Pathway enrichment analysis was performed using Enrichr on differentially expressed genes (DEGs) identified from *Cpt1a*flox MHV-68 and *Cpt1a Spc*-KO in intermediate 2 cells (Figure 5E).

Pathway analysis of single cell fixed RNA sequencing data from healthy donors (*n* = 13) and IPF patients (*n* = 6) was used to predict cholesterol biosynthesis, along with other pathways such as regulation of cholesterol biosynthesis, metabolism, mitochondrial biogenesis, and fatty acid oxidation in the Reactome 2022 database (Supplementary Figure 1B).

Score analysis

To normalize the gene expression data, z-scores were calculated using a custom function, calculate_z_score, which utilizes the mean and sd functions from R. This normalization process allowed for the comparison of relative gene expression across different cell groups.

Xenium In Situ

Gene Panel Design

Xenium in situ technology relies on a pre-defined gene panel. Each probe consists of two complementary sequences targeting the mRNA of interest and a unique gene-specific barcode. Upon hybridization, the probe circularizes and undergoes rolling circle amplification, amplifying the signal for target detection and decoding. A total of 389 genes were analyzed using Chemistry version v1. Of these, 289 genes were derived from a Xenium human lung gene expression panel by 10X genomics, while 100 genes from an add-on custom designed panel (CH7CY9 by Mora&Rojas Lab). The custom panel was curated based on human lung single-cell analysis data generated by the Mora & Rojas Lab, focusing on genes relevant for cell type identification and potential involvement in IPF.

Cell Segmentation

The Xenium In Situ Cell Segmentation Kit, PN- 1000662 (10X genomics) was used to improve the determination of cell boundaries by using a stain and algorithmic technique developed and validated through custom-trained machine learning models.

Sample preparation

FFPE-lung tissue was sectioned 5x 6 mm at 5 µm onto a Xenium slide. Sections were treated to access the RNA for labeling with circularizable DNA probes. Probe ligation generated a circular DNA probe that was enzymatically amplified. Slides were placed in the Xenium Analyzer (Instrument serial number XETG00239) where the sample underwent successive rounds of fluorescent probe hybridization, imaging, and removal; creating bright, easy to image signal with a high signal-to-noise ratio. An optical signature specific to each gene was generated, enabling target gene identification. Finally, a spatial map of the transcripts in the entire tissue section was built using Xenium analysis software version -2.0.0.10 and instrument software version 2.0.1.0, which allowed immediate exploration of the assay's subcellular readout. This functionality is delivered in two parts: (1) comprehensive onboard analysis and (2) off-instrument analysis that leverages the exploration-ready output generated by the Xenium Analyzer.

The assignment of transcripts to cells, precision of specific transcript locations and checking cell segmentation were performed in the final step of the analysis workflow in Xenium Explorer v3.0 software (interactive visualization tool by 10X genomics).

Detailed instructions on consumable preparation and instrument operation can be found in 10X Genomics' User Guide CG000749, Rev A.

Quantification

To quantify SFTPC+ cells and associated gene expression from Xenium, a sampling approach was employed to acquire 5 random fields of view (FOV) from each tissue slide. The FOV sizes were identical in every tissue specimen and specifically chosen to cover

sufficient numbers of cells but not sample more than 60% of the overall tissue. Prior to extraction, the Xenium data was processed using Seurat v5 and normalized using SCTransform. Segmentation of individual cells and attribution of gene probes was carried out directly on the Xenium analyzer and all default values were utilized. SFTPC+ cells were identified in each sample as cells having 2 or more counts for the SFTPC probe. Over 60% of cells, on average, had no counts for the probe, but selecting 2 counts instead of 1 will account for any wrongly attributed probes due to segmentation error. Within each FOV, average normalized gene expression values for selected genes were captured from cells labeled as SFTPC+.

Statistics

Statistical differences between two groups were compared using Student's t-test. All other statistical analyses with more than two groups were determined by one-way or two-way ANOVA. Values of $p < 0.05$ were considered significant. Data are represented as individual dots ± standard deviation and replicates are as indicated in figure legends. Statistical analyses were performed using Graph Pad Prism software (version 10.1.2).

Study approval: Animal use was approved by the IACUC at University of Pittsburgh and adhered to NIH guidelines for the use of experimental animals. Human lung tissues were collected from excess pathologic tissues after lung transplantation and organ donation, under University of Pittsburgh Institutional Review Board and CORID-approved protocols (970946, PRO14010265, and CORID No. 300). Demographics data from samples used in this study can be found in Supplemental Table S1.

References

1. Bueno M, Brands J, Voltz L, Fiedler K, Mays B, St Croix C, et al. ATF3 represses PINK1 gene transcription in lung epithelial cells to control mitochondrial homeostasis. *Aging Cell.* 2018;17(2).
2. Calyeca J, Balderas-Martínez YI, Olmos R, Jasso R, Maldonado V, Rivera Q, et al. Accelerated aging induced by deficiency of Zmpste24 protects old mice to develop bleomycin-induced pulmonary fibrosis. *Aging (Albany NY).* 2018;10(12):3881-96.
3. Bueno M, Lai YC, Romero Y, Brands J, St Croix CM, Kamga C, et al. PINK1 deficiency impairs mitochondrial homeostasis and promotes lung fibrosis. *J Clin Invest.* 2015;125(2):521-38.
4. Mora AL, Woods CR, Garcia A, Xu J, Rojas M, Speck SH, et al. Lung infection with gamma-herpesvirus induces progressive pulmonary fibrosis in Th2-biased mice. *Am J Physiol Lung Cell Mol Physiol.* 2005;289(5):L711-21.
5. Korfei M, Ruppert C, Mahavadi P, Henneke I, Markart P, Koch M, et al. Epithelial endoplasmic reticulum stress and apoptosis in sporadic idiopathic pulmonary fibrosis. *Am J Respir Crit Care Med.* 2008;178(8):838-46.
6. Chen Q, and Liu Y. Isolation and culture of mouse alveolar type II cells to study type II to type I cell differentiation. *STAR Protoc.* 2021;2(1):100241.
7. Jacob A, Morley M, Hawkins F, McCauley KB, Jean JC, Heins H, et al. Differentiation of Human Pluripotent Stem Cells into Functional Lung Alveolar Epithelial Cells. *Cell Stem Cell.* 2017;21(4):472-88 e10.
8. McCauley KB, Alysandratos KD, Jacob A, Hawkins F, Caballero IS, Vedaie M, et al. Single-Cell Transcriptomic Profiling of Pluripotent Stem Cell-Derived SCGB3A2+ Airway Epithelium. *Stem Cell Reports.* 2018;10(5):1579-95.
9. Hawkins F, Kramer P, Jacob A, Driver I, Thomas DC, McCauley KB, et al. Prospective isolation of NKX2-1-expressing human lung progenitors derived from pluripotent stem cells. *J Clin Invest.* 2017;127(6):2277-94.
10. Burgess CL, Huang J, Bawa PS, Alysandratos KD, Minakin K, Ayers LJ, et al. Generation of human alveolar epithelial type I cells from pluripotent stem cells. *Cell Stem Cell.* 2024;31(5):657-75 e8.
11. Huynh FK, Green MF, Koves TR, and Hirschey MD. Measurement of fatty acid oxidation rates in animal tissues and cell lines. *Methods Enzymol.* 2014;542:391-405.
12. Bligh EG, and Dyer WJ. A rapid method of total lipid extraction and purification. *Can J Biochem Physiol.* 1959;37(8):911-7.
13. Crotta S, Villa M, Major J, Finsterbusch K, Llorian M, Carmeliet P, et al. Repair of airway epithelia requires metabolic rewiring towards fatty acid oxidation. *Nat Commun.* 2023;14(1):721.
14. Wang F, Ting C, Riemondy KA, Douglas M, Foster K, Patel N, et al. Regulation of epithelial transitional states in murine and human pulmonary fibrosis. *J Clin Invest.* 2023;133(22).
15. Zheng GX, Terry JM, Belgrader P, Ryvkin P, Bent ZW, Wilson R, et al. Massively parallel digital transcriptional profiling of single cells. *Nat Commun.* 2017;8:14049.
16. Hafemeister C, and Satija R. Normalization and variance stabilization of single-cell RNA-seq data using regularized negative binomial regression. *Genome Biol.* 2019;20(1):296.
17. Takeuchi A, and Onodera K. Effects of kainic acid on the glutamate receptors of the crayfish muscle. *Neuropharmacology.* 1975;14(9):619-25.
18. Han S, Lee M, Shin Y, Giovanni R, Chakrabarty RP, Herrerias MM, et al. Mitochondrial integrated stress response controls lung epithelial cell fate. *Nature.* 2023;620(7975):890-7.
19. Gu Z, Eils R, and Schlesner M. Complex heatmaps reveal patterns and correlations in multidimensional genomic data. *Bioinformatics.* 2016;32(18):2847-9.
