# Supplementary figures and images for: Regulation of lung progenitor plasticity and repair by fatty acid oxidation
Source: JCI Insight. 2025 Feb 10;10(3):e165837. doi: 10.1172/jci.insight.165837 (PMC11948574; doi:10.1172/jci.insight.165837)

Full unedited gel for Figure 7 A

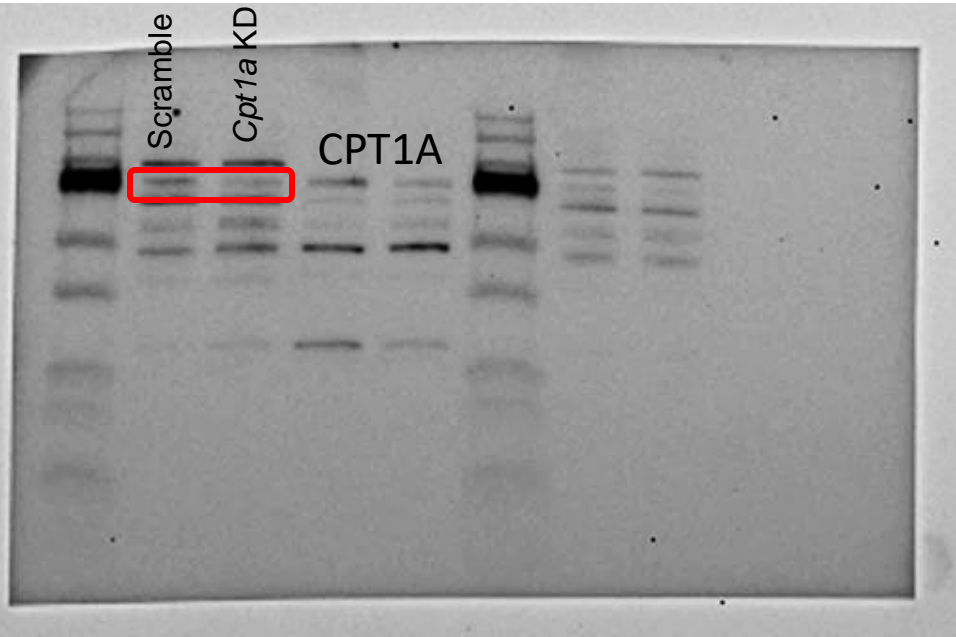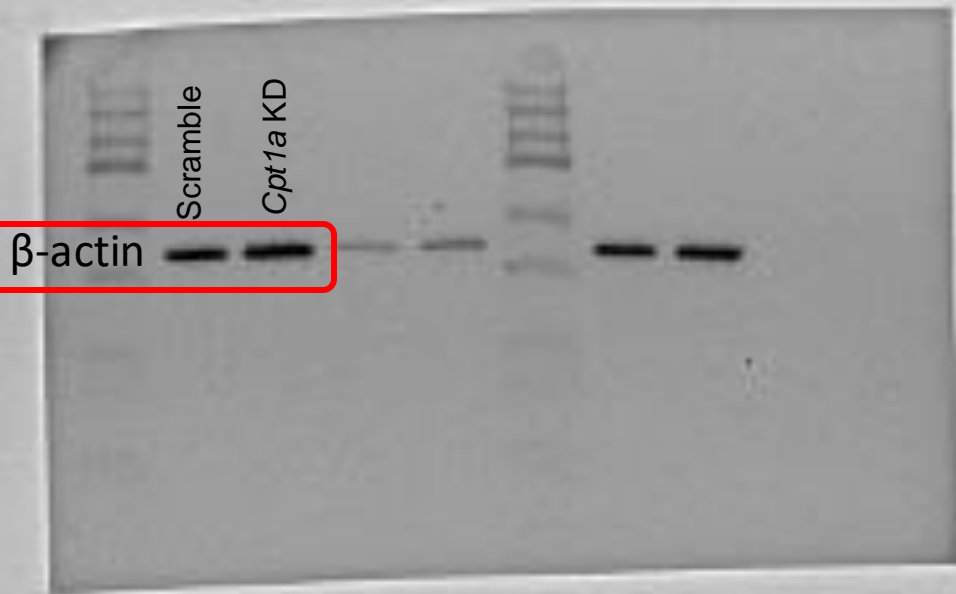

Full unedited gel Figure 8 F

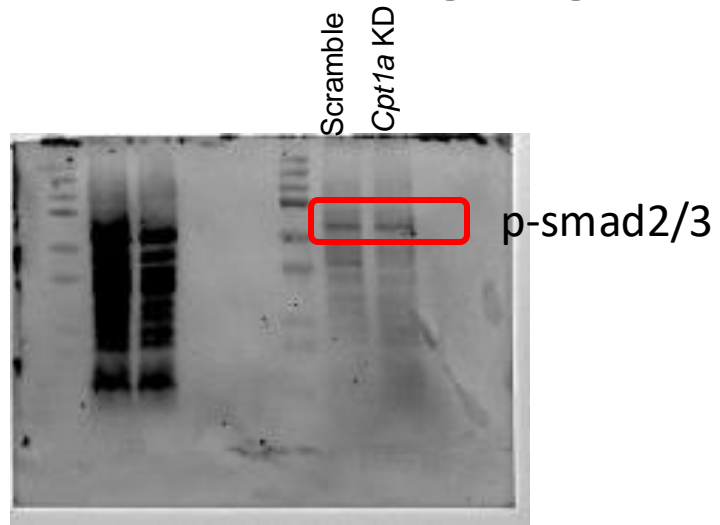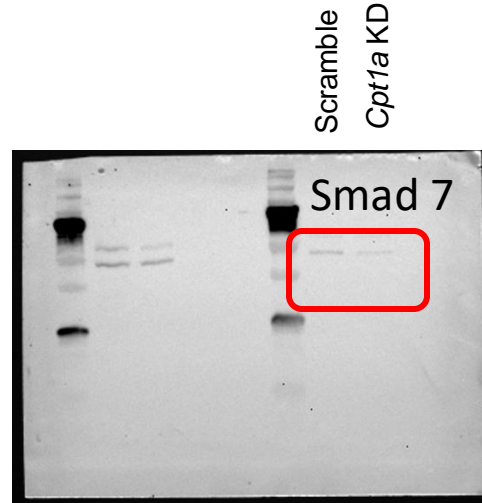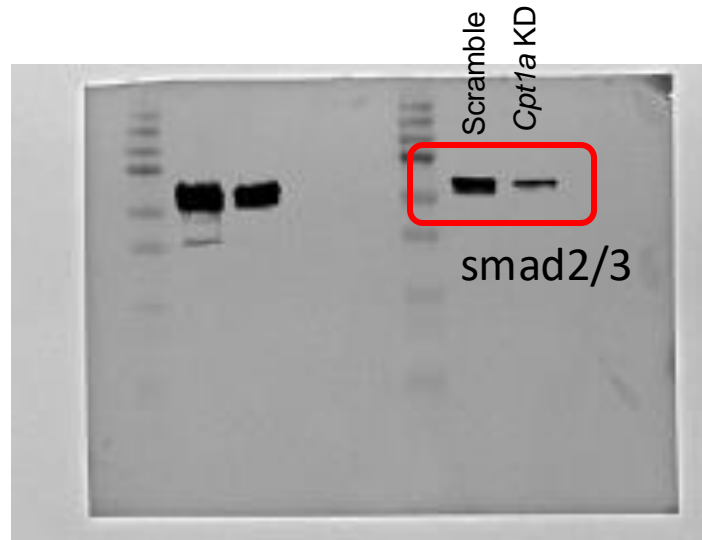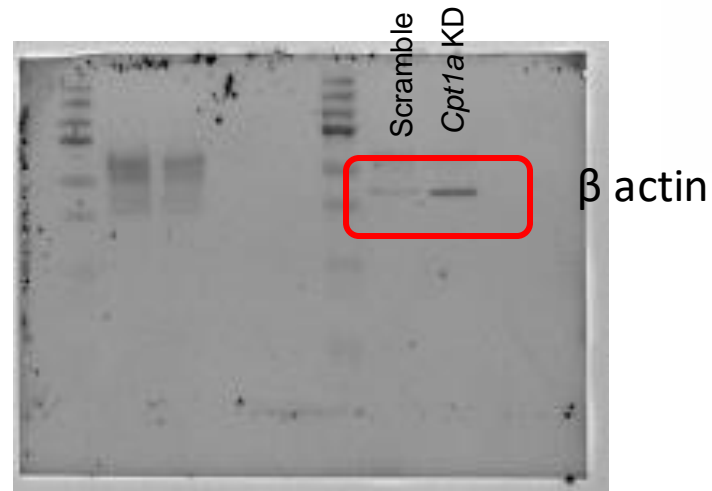

Full unedited gel Figure 8 G

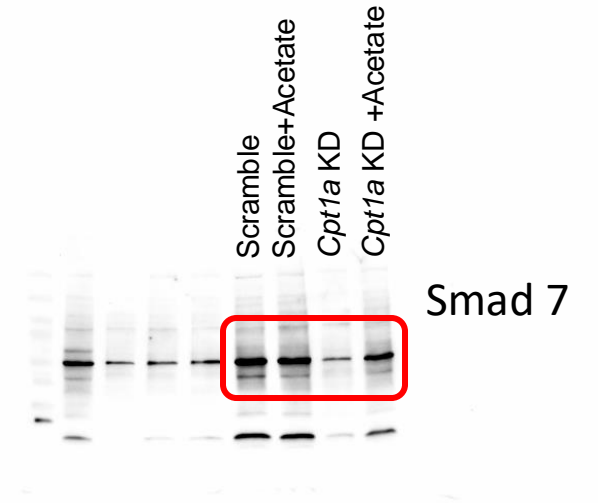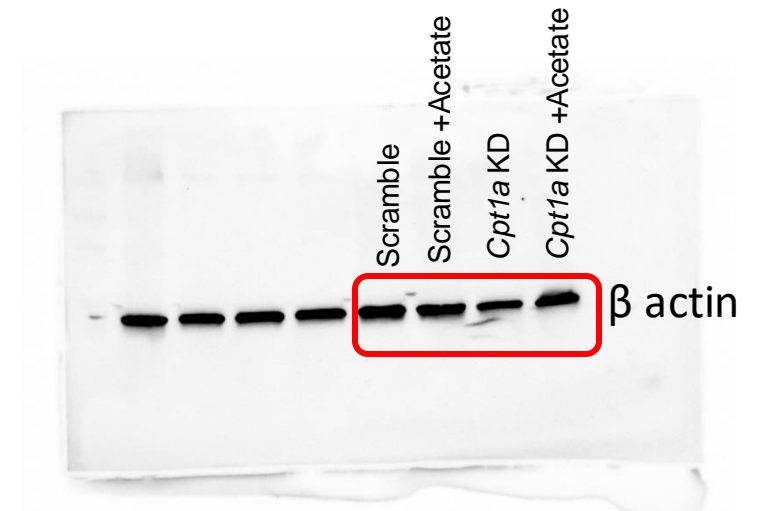

Supplement: Unedited blot and gel images [file jciinsight-10-165837-s075.pdf]
